# Microtubule-dependent regulation of chromatin dynamics by the Smc5/6 complex

**DOI:** 10.1016/j.isci.2026.116943

**Published:** 2026-08-03

**Authors:** Ànnia Carré-Simon, Cecilia Martuzzi, Sarah Isler, Renaud Batrin, Henrik Dahl Pinholt, Timothy Földes, Guillaume Laflamme, Juan Reguera, Maria Barbi, Leonid Mirny, Damien D’Amours, Emmanuelle Fabre

**Affiliations:** 1 Université Paris Cité, INSERM, CNRS, IRSL, UMR1342, EMR8000, Paris, France; 2 Ottawa Institute of Systems Biology, Department of Cellular and Molecular Medicine, Ottawa, ON, Canada; 3 Department of Physics, Massachusetts Institute of Technology, Cambridge, MA, USA; 4 Sorbonne Université, CNRS, LPTMC, Paris, France; 5 Université -Marseille, CNRS, UMR7257, Marseille, France; 6 Institute for Medical Engineering and Science, Massachusetts Institute of Technology, Cambridge, MA, USA

**Keywords:** Smc5/6, microtubules, pericentromeres, mitotic spindle, DNA double-strand break repair, chromatin dynamics

## Abstract

Proper centromeres and pericentromeres folding is crucial for maintaining genome integrity and ensuring accurate chromosome segregation. Smc5/6 complex, which localizes at pericentromeres, can bind microtubules, yet its role in chromatin folding is unclear. Here, we investigate the functional relevance of Smc5/6 binding to microtubules in yeast using a separation-of-function mutant (*smc5-2KE*) that disrupt this interaction but leaves other biochemical activities of the complex intact. We demonstrate that Smc5/6 binding to microtubules constrains chromatin dynamics and regulates pericentromeric chromatin organization, as revealed by high-temporal-resolution imaging, polymer modeling, and *in vitro* approaches. Weakening of kinetochore activity in *smc5* mutant cells leads to significant spindle defects and triggers the spindle checkpoint. Ultimately, the inability of Smc5/6 to associate effectively with microtubules and regulate pericentric chromatin compromise homologous recombination repair of DNA damage near centromeres. Overall, our findings indicate that Smc5/6—microtubules association preserves pericentromeric chromatin architecture and genome stability during mitosis.

## Highlights

• Smc5/6 binding to microtubules constrains chromatin dynamics
• Smc5/6-microtubule association regulates pericentromeric organization
• Weakening this association causes spindle defects and checkpoint activation
• Smc5/6-microtubule association promotes DNA repair near centromeres

## Graphical abstract

**Figure:**
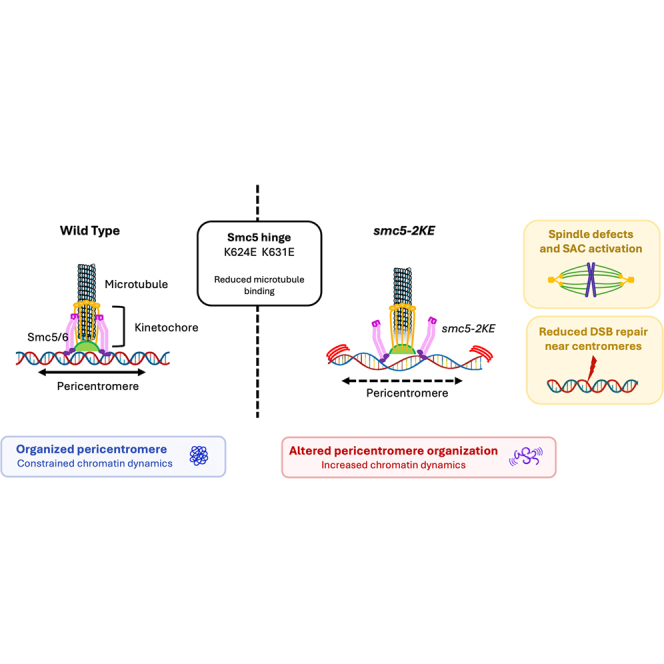

## Teaser

Biological sciences

## Introduction

The three-dimensional architecture of the genome governs gene expression, DNA replication and repair.^1^ Centromeres are specialized regions within this chromosomal architecture that serve as scaffolds for microtubule attachment via the kinetochore, a complex of around 80 proteins.^2^^,^^3^ Surrounding the centromere, pericentromeric regions adopt a distinct chromatin configuration that is essential for genome stability.^4^ Pericentromeric configuration and centromere/kinetochore assembly are crucial to counterbalance the mechanical forces exerted by spindle microtubules during mitosis. Consistently, increased mobility of pericentromeric chromatin is observed when centromeres are released from their attachment to microtubules.^5^^,^^6^^,^^7^^,^^8^ Yet, how pericentromeric structure is shaped to resist the forces exerted by microtubules is poorly understood.^9^^,^^10^ In budding yeast, pericentromeres span around 30–50 kb^11^ and are highly enriched in structural chromosome maintenance (SMC) complexes, which include cohesins, condensins and the Smc5/6 complex (Smc5/6 in short).^12^^,^^13^^,^^14^^,^^15^ Cohesins and condensins participate in the establishment of DNA loops that resist to mitotic spindle forces.^16^ While assembly of an Smc5/6 dimer was recently shown to contribute to DNA loop folding *in vitro,*^17^ the folding function of Smc5/6 at pericentromeres is unknown.

Smc5/6 is composed of Smc5, Smc6 and non-SMC proteins (Nse1, Mms21/Nse2 and Nse3-6).^18^ Smc5 and Smc6 consist of antiparallel coiled-coil arms that dimerize via a hinge domain at one end and are linked at the other end by their ATPase head domains. As a key player in genome stability that mediates post-translational modifications (PTMs), Smc5/6 interacts with many chromosome proteins during its cellular functions.^18^ One of the most intriguing interactors of the complex is the alpha/beta tubulin dimer. We previously showed *in vitro* that the hinge domain of the Smc5 protein binds the tubulin dimer exclusively in the form of microtubule polymers.^19^ The hinge domain contains three lysines (K624, K631, and K667) that interact directly *in vitro* with the C-terminal negatively charged tails of tubulins.^19^ A recent mass spectrometry analysis of overexpressed Smc5/6 purified from exponentially growing yeast also identified alpha/beta tubulin subunits Tub1 and Tub2 as cofactors binding to the Smc5/6 complex,^20^ an observation that we have independently reproduced (Figures S1A–S1C). These data support an interaction between the Smc5/6 complex and microtubules, but the function of such a link remains an open question.

Besides its enrichment at pericentromeres, Smc5/6 is also found at telomeres and rDNA to maintain their organization.^21^^,^^22^^,^^23^ For example, deletion of the Nse2 (Mms21) subunits of Smc5/6 leads to defects in genome organization, including telomere declustering and nucleolar fragmentation.^21^^,^^22^^,^^23^ Additionally, an Smc5/6 mutant (*smc6-9*) exhibits chromosome segregation defects, notably in repetitive regions such as rDNA and telomeres.^22^^,^^24^ Beyond its structural role, Smc5/6 participates in genome integrity by promoting the repair of damaged DNA.^18^^,^^25^ Smc5/6 is recruited within ∼5 kb of double-strand breaks (DSBs),^26^^,^^27^ holds the DSB ends together^28^ and facilitates homologous recombination (HR),^22^^,^^29^^,^^30^^,^^31^ a DNA repair process that uses a homologous sequence as a template to accurately repair DSBs.^32^

Here, we examine how Smc5/6-microtubule association regulates pericentromeric chromatin dynamics, combining mutational analysis, live-cell fluorescence microscopy and polymer simulations. Our findings reveal that this interaction impacts pericentromeric chromatin folding, dynamics and integrity.

## Results

### The *smc5-2KE* mutant leads to increased chromatin dynamics at pericentromeres

**Figure 1:**
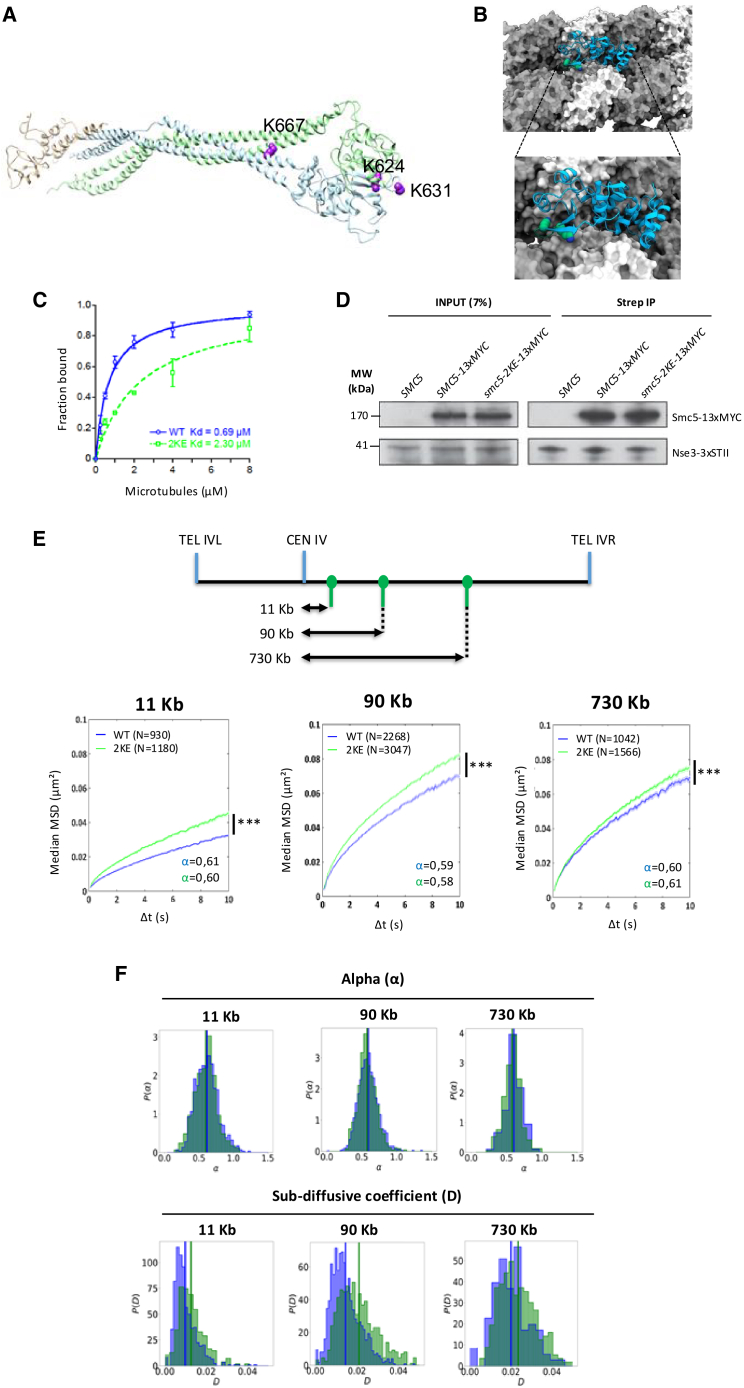
The *smc5-2KE* mutant shows decreased binding affinity to microtubules *in vitro* and leads to increased chromatin dynamics at pericentromeres and beyond (A) Representation of the Cryo-EM of the Smc5-Smc6 hinge from^4^ (PDB: 7YLM). The Smc5 hinge domain is shown in blue, the Smc6 hinge in green, and Nse2 in brown. Lysines 624, 631, and 667 are highlighted in purple. (B) AlphaFold model of the Smc5 hinge (amino acids 450–645, cartoon in light blue) showing a high predicted probability of interaction with α/β tubulin. α-tubulin (Tub1) and β-tubulin (Tub2) are shown in light and dark gray, respectively. Highlighted the basic side chains of Lysines 624 and 631 (4 green balls) and the C-loop (green). The highly reactive amino groups of K624 and K631 are shown in dark blue and contact adjacent tubulin subunits in the microtubule. Bottom pannel is an enlargement of the top one. (C) The fraction of microtubule-bound Smc5 and Smc5-2KE from 3 independent experiments was plotted as a function of microtubule (MT) concentrations. Dissociation constants (Kd) were determined by curve fitting to a hyperbola. (D) Co-immunoprecipitation (co/IP) experiments show the binding of Nse3 tagged with C-terminal tandem Strep-tag II (3xSTII) to Smc5 or Smc5-2KE proteins (both tagged with C-terminal 13xMYC epitopes) in yeast extracts. A whole cell extract was loaded as an input (7%; left) and Smc5/6 complex components were immunoprecipitated from this extract using StrepTactin XT resin (right). For each panel, the first lane represents a negative control where only Nse3 was tagged (with 3xSTII), whereas the second and third lanes show Nse3-3xSTII co-purified with Smc5-13xMYC and Smc5-2KE-13xMYC proteins, respectively. (E) Schematic representation of chromosome IV showing the position of the three GFP-labeled chromatin arrays relative to the centromere (CEN) (top). Mean square displacement (MSD) as a function of increasing time intervals in the WT (blue) and in the *smc5*-*2KE* mutant (green) for a locus at different positions of chromosome IV (11 kb, 90 kb and 730 kb, from left to right). Statistical significance was assessed using a two-sided Wilcoxon rank-sum test; ∗*p* < 0.05 and ∗∗∗*p* < 0.0001. (bottom). (F) Distributions of the exponent alpha (upper line) and of the sub-diffusion coefficient D (lower line) for the WT (blue) and in the *smc5-2KE* mutant (green) for loci at different positions of chromosome IV. The values of parameters alpha and D are calculated from single trajectories as described in Materials and Methods. Vertical lines correspond to average values (WT in blue, *smc5-2KE* mutant in green).

To determine how Smc5/6 and microtubules could interact, we analyzed the spatial distribution of K624, K631 and K667 residues in the hinge domain (PDB:7YLM^33^), using the USCF Chimera program. The structural model revealed that K624 and K631 are positioned on the outer surface of the hinge domain, making them accessible for potential interactions with microtubules. In contrast, K667 is embedded in the coiled-coil arm of Smc5 and could not theoretically interact with microtubules (Figure 1A). These results agree with the position of K624 and K631 in the specific loop of the C domain unique to the Smc5/6 complex observed in the X-ray crystal structures of the *S. pombe* Smc5 hinge domain.^34^

In a second step, using AlphaFold3 to model alpha/beta tubulin subunits and a segment of the Smc5 hinge domain containing K624 and K631, we identified a high probability of interaction between this region and Tub1/Tub2 dimer (iPTM = 0.72) revealing an interaction interface at the surface of the assembled microtubules (Figure 1B), in contrast with the low probability of interaction with other proteins such as the subunit Shs1 of the septin complex (iPTM = 0.18) (Figures S1D and S1E).

This observation led us to focus on K624 and K631 and modify their electrostatic attraction with the C-terminal tubulin tails by mutating them to negatively charged glutamic acid residues, generating the *smc5-2KE* mutant (STAR Methods; Table 1 and^19^). The microtubule-binding capacity of the *smc5-2KE* mutant was verified *in vitro* by comparing the co-sedimentation of purified Smc5 and Smc5-2KE proteins with microtubules. We observed a 3-fold reduction in the binding affinity of Smc5-2KE compared to wild-type (WT) Smc5 (Figures 1C and S1F). The reduced affinity of Smc5-2KE for microtubules was not due to a defect in the mutant’s interaction with other proteins in the Smc5/6 complex, as indicated by co-immunoprecipitation (CoIP) of the Nse3 subunit with both Smc5 and Smc5-2KE (Figure 1D). The preservation of Smc5/6 integrity in the *smc5-2KE* mutant is consistent with the absence of detectable defects in growth, cell division, cell cycle progression, DNA damage sensitivity, or thermotolerance in this mutant, in the BY background used for our chromatin and HR assays (Figures S2A–S2G). Notably, we find that sensitivity of *smc5-2KE* to 4NQO and HU is strain-background dependent, with clear drug sensitivity observed in a W303 background but not in BY under the conditions tested (Figure S2F). Furthermore, the *smc5-2KE* mutant did not impact the binding of the protein to DNA, as observed by ChIP-qPCR at centromeres, telomeres, and rDNA (Figure S2H), indicating that the associations of Smc5/6 with microtubules and with chromatin are independent. Therefore, we used *smc5-2KE* as a separation-of-function mutant that is specifically defective in microtubule binding to explore the role of Smc5/6 association with microtubules in chromatin folding and dynamics *in vivo*.

To first assess how reduced microtubule-Smc5/6 association affects chromatin dynamics as a function of chromosome position, we tracked a GFP-labelled chromatin array (TetO-TetR-GFP) positioned at 11, 90, and 730 kb from centromere IV (CEN IV) in both WT and *smc5-2KE* mutant. We calculated the mean square displacement (MSD) as previously described and extracted different dynamic parameters^5^^,^^35^^,^^36^^,^^37^^,^^38^^,^^39^^,^^40^ (Materials and methods). In WT cells, the different tracked loci followed a sub-diffusive movement characterized by an anomalous *α* exponent below 1.0 (between 0.59 and 0.61, Figures 1E and S3A). These *α* values are consistent with predictions from self-avoiding bead-spring polymer models^41^ and suggest that the links between nucleosomes, which qualitatively determine the nature of chromosome dynamics, are independent of distances from the centromere. However, the sub-diffusive coefficient D, as well as MSD at 10s (MSD_10s_), indicated higher mobility at positions further from the centromeric attachment point to kinetochore microtubules on CEN IV, as previously documented (Figures 1E, 1F, and S3A–S3C).^6^^,^^36^^,^^37^^,^^38^^,^^42^ Interestingly, *smc5-2KE* mutated cells maintained sub-diffusive chromatin with *α* values such as WT, but displayed increased chromatin dynamics, characterized by higher diffusion coefficients (D), at all examined loci. An increase in D can be explained by reduced interactions within the chromatin polymer or with external factors, consistent with the loss of centromeric constraint and/or partial detachment of centromeric chromatin from kinetochore microtubules.^43^ Notably, the increase in chromatin dynamics in mutated cells was more pronounced at the locus proximal to the centromere (Figures 1E and 1F). Lastly, MSD_10s_ values increased in the *smc5-2KE* mutant, compared to WT, by factors of 1.36, 1.18 and 1.07 for distances of 11, 90, and 730 kb from CEN IV, respectively (Figure S3C). Increased chromatin mobility of the *smc5-2KE* mutant was verified for another locus located at 90 kb from CEN V (Figure S3D) and observed at different phases of the cell cycle, including G1 and metaphase (Figure S4).

Lysine residues undergo a wide range of reversible PTMs such as SUMOylation, acetylation, and ubiquitination, which can regulate protein functions, including protein-protein interactions.^44^ To determine whether PTM of K624 and K631 contributes to the Smc5 effect on chromatin mobility, we substituted both lysines with arginines, preserving the positive charges while preventing PTM. The *smc5-2KR* mutant showed chromatin mobility similar to WT (Figure S3D), indicating that the positive charges at amino acid positions 624 and 631 of Smc5, rather than their potential to be modified by PTMs, are required to promote normal chromatin dynamics *in vivo*.

Finally, since sister chromatid cohesion restricts chromatin dynamics during S phase,^45^^,^^46^ we investigated whether cohesion defects in the *smc5-2KE* mutant could account for the observed changes in chromatin dynamics by analyzing TetO-TetR-GFP at 11 kb from CEN IV in mitotically synchronized cells. We quantified the number of cells displaying either one GFP focus (cohesive chromatin) or two GFP foci (defective cohesion).^11^^,^^47^ Our analysis revealed no significant differences in the number of cells with single GFP foci between the *smc5-2KE* mutant and the WT strain (Figure S3E), indicating no cohesion defects. In contrast, the deletion of the Smc1 cohesin subunit, used as a control for defective cohesion, resulted in the separation of GFP foci (Figure S3F).^47^

Altogether, our results support the hypothesis that K624 and K631, which are conserved in the C domain of Smc5 hinge, limit chromatin dynamics possibly through the attraction of their positive charge to microtubules, as observed *in vitro*.

### Increased chromatin dynamics of the *smc5-2KE* mutant is not linked to spontaneous DNA damage or loss of Smc5/6

Increase in chromatin dynamics is part of the DNA Damage Response.^48^^,^^49^ To determine whether higher chromatin mobility in the *smc5-2KE* mutant could result from spontaneous DNA damage, we monitored Rad52 foci damage,^50^ Rad53 phosphorylation,^51^ and histone H2A phosphorylation (γH2A).^52^ *smc5-2KE* cells showed low levels of Rad52 foci, Rad53 phosphorylation, and γH2A comparable to WT, indicating that the increased chromatin dynamics observed in the *smc5-2KE* mutant are not explained by high levels of spontaneous DNA damage (Figure S5).

While K624E and K631E mutations do not affect the stability of Smc5/6 *in vitro* (Figure 1D),^50^^,^^51^^,^^52^^,^^53^ it remains possible that a defect in the Smc5/6 complex, undetectable *in vitro*, could account for the change in chromatin mobility of the *smc5-2KE* mutant observed *in vivo*. To address this point, we investigated chromatin mobility in cells defective for Smc5/6 by specifically degrading Smc5 or Smc6 proteins using the auxin-inducible degron (AID) strategy. Cells expressing AID-tagged Smc5 or Smc6 proteins grew such as WT cells in the absence of auxin but failed to grow in the presence of 1 mM auxin (Figures S6A and S6B). This indicates efficient degradation of the tagged proteins and confirms that loss of Smc5 or Smc6 proteins is incompatible with cell viability, unlike the *smc5-2KE* mutant, which has no growth defect. Degradation took place 30 min after auxin addition, as shown by a western blot for Smc5-AID (Figure S6C). Surprisingly, a reduction in chromatin dynamics in the pericentromeric region, at 11 kb from the centromere, was observed when Smc5-AID or Smc6-AID protein degradation was induced by 1 h of auxin addition (Figures S6D–S6F). These findings contrast with the increase in chromatin dynamics observed upon degradation of the cohesin subunit Smc1 (Figures S6E and S6F), as previously documented.^6^^,^^46^^,^^54^^,^^55^ The opposite effects of Smc5/6 and cohesin on pericentromeric chromatin dynamics suggest that these complexes likely act via distinct mechanisms. While cohesin’s role in loop extrusion may explain its effect on chromatin mobility, the mechanism by which the full Smc5/6 complex maintains chromatin dynamics remains to be fully elucidated.

### The binding of microtubules to kinetochores is necessary but not sufficient to control pericentromeric chromatin dynamics

**Figure 2:**
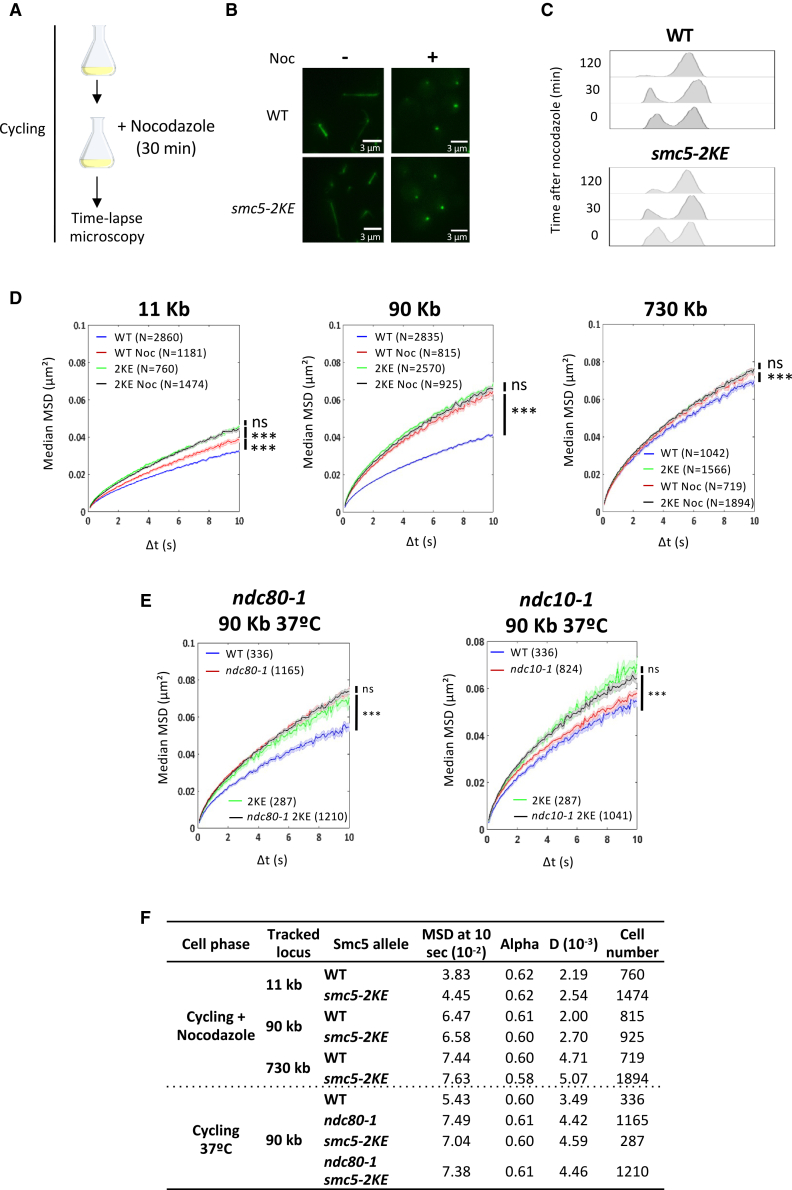
The binding of microtubules to the kinetochore is necessary but not sufficient to control pericentromeric chromatin dynamics (A) Experimental protocol used to depolymerize microtubules with 15 ng/μL of nocodazole while continuing cycling. (B) Tub2-GFP in the WT and in the *smc5-2KE* mutant with or without addition of nocodazole. Scale bar, 3 um. (C) Cell synchronization monitored by flow cytometry in cells treated with nocodazole (15 ng/μL). Samples were taken at 0, 30 and 120 min after nocodazole addition. (D) MSD as a function of increasing time intervals in the WT and *smc5*-2KE mutant at 11, 90 and 730 kb with or without the presence of nocodazole. Wilcoxon rank-sum test results between distributions, with the *p* value “ns” non-significant, ∗∗∗ *p* < 0.0001. (E) MSDs as a function of increasing time intervals of the locus at 90 kb in the *ndc80-1* mutant (left) and *ndc10-1* mutant (right) combined with or without the *smc5-2KE* mutation at 37°C. Statistical significance was assessed using a two-sided Wilcoxon rank-sum test; ns, not significant; ∗∗∗*p* < 0.0001. (F) Table summarizing the different conditions with values extracted from the MSD: MSD at 10 s, the anomalous exponent (alpha), the sub-diffusive coefficient (D) and the number of cells analyzed.

The constraint of pericentromeric chromatin dynamics by Smc5/6 is in line with findings in budding yeast, where releasing centromeres from their attachment to the spindle pole body (SPB)—either upon microtubule depolymerization or centromere inactivation—increases chromatin mobility.^5^^,^^6^^,^^7^^,^^8^ We therefore examined chromatin mobility following microtubule depolymerization by nocodazole in WT and in the *smc5-2KE* mutant. We exposed cells to a short nocodazole treatment (30 min) (Figure 2A) and analyzed chromatin dynamics at different chromosome locations. This treatment depolymerized microtubules, as confirmed by the absence of microtubule filaments visualized by Tub2-GFP (Figure 2B) but did not block the cell cycle (Figure 2C). In WT cells, chromatin mobility increased upon exposure to nocodazole at loci far from the centromere, i.e., at 90 kb and 730 kb from the centromere, as previously observed (Figure 2D, middle and right panels).^5^^,^^7^ In contrast, in the *smc5-2KE* mutant, nocodazole had no significant effect on chromatin mobility, indicating no cumulative effect of the two. Close to the centromere, at 11 kb from CEN IV, the increase in chromatin dynamics caused by nocodazole was lower than that observed in the *smc5-2KE* mutant (MSD_10s_ compared to WT, 1.14 vs. 1.32, respectively, Figure 2D, left panel). These observations suggest that binding of microtubules to Smc5/6 restricts chromatin mobility throughout the chromosome and that in the pericentromeric region, Smc5/6 plays an additional role, possibly affecting chromatin properties.

In a complementary approach, we probed chromatin dynamics in two kinetochore thermosensitive mutants, *ndc80*-1, which impairs kinetochores-microtubules binding^56^ and *ndc10*-1, which affects centromeric chromatin binding.^57^ To effectively compare these mutants to *smc5-2KE*, we first confirmed that the chromatin behavior reported in the *smc5-2KE* mutant at 30°C was also observed at 37°C, the restrictive temperature for these mutants. Chromatin dynamics at 90 kb from CEN V displayed a comparable increase between *ndc80-1* and *smc5-2KE* single mutants at 37°C (Figures 2E left and 2F), with no additive effect in the *ndc80-1 smc5-2KE* double mutant (Figure 2E left, F). Conversely, the *ndc10-1* mutant showed no increase in chromatin dynamics compared to the WT, and an increase in chromatin mobility when combined with the Smc5-2KE mutation (Figures 2E right and 2F). These results indicate that only kinetochore mutations disrupting microtubule binding and not chromatin binding mimic the *smc5-2KE* mutant.

Our data collectively demonstrate that changes in microtubule binding, either due to *smc5-2KE*, kinetochore mutants that impair binding with microtubules, or microtubule depolymerization, are sufficient to enhance chromatin dynamics of loci at a distance of 90 kb from the centromere. They also indicate that Smc5/6 plays an additional role in controlling pericentromeric dynamics through its binding to microtubules.

### The *smc5-2KE* mutant modifies pericentromeric chromatin properties

**Figure 3:**
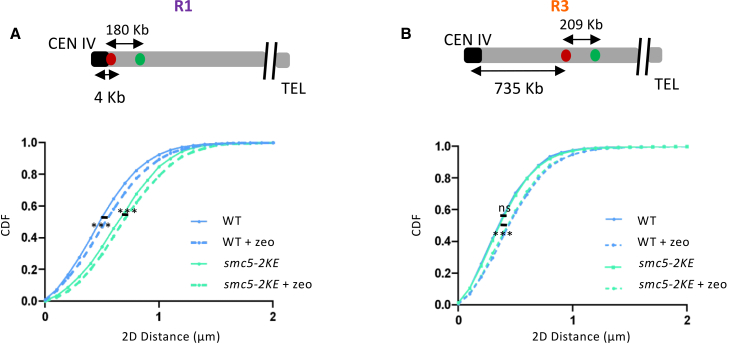
The *smc5-2KE* mutant changes chromatin properties in the pericentromeric region (A and B) Cumulative distribution functions (CDF) show the intrachromosomal distances distribution measured for two pairs of loci 180 kb apart at two different positions of chromosome IV, (A) close to the centromere (R1) and (B) in the middle of the arm (R3) in the presence or absence of Zeocin. Three experiments were done in each case: R1: N_WT_ = 21806, N_2KE_ = 9014, N_WT ZEO_ = 4091, N_2KE ZEO_ = 1570 and for R3: N_WT_ = 2778, N_2KE_ = 4403. Statistical significance was assessed using a two-sided Wilcoxon rank-sum test; ns, not significant, ∗∗∗*p* < 0.001.

To better understand the impact of the *smc5-2KE* mutation on chromatin properties, we compared intra-chromosomal distances of different loci in WT and mutant cells, as changes in intra-chromosomal distances reflect changes in compaction, rigidity or loop extrusion activity.^28^^,^^38^^,^^58^ We used strains R1 and R3 where pairs of differently fluorescently labeled loci, distant by ∼180 kb, are scattered on chromosome IV^38^ (Material and methods). The R1 strain has two labeled loci, one in a pericentromeric position (at 4 kb from CEN IV) and one outside (Figure 3A), and the R3 strain has two labeled loci located centrally on the chromosome arm (at 735 kb from CEN IV, Figure 3B). The Smc5-2KE mutation was introduced in both strains, and 2D distances between loci were measured from 3D images (Materials and methods). As a control, we used Zeocin, which promotes an increase in intra-chromosomal distances at all positions, as previously shown (Figures 3A and 3B).^38^^,^^58^ In the R3 strain, intra-chromosomal distances between the loci were similar in R3 WT and R3 *smc5-2KE* (Figure 3B). In contrast, the R1 *smc5-2KE* mutant displayed a significant increase in distance compared to R1 WT (Figure 3A). This increase suggests that at this sub-chromosomal scale, changes in chromatin properties occur in the *smc5-2KE* mutant, which are revealed when at least one locus is pericentromeric. Although these data do not precisely define whether chromatin decompaction or stiffening are induced by the *smc5-2KE* mutant, they do indicate that the K624- and K631-dependent association of Smc5 with microtubules is required to establish or maintain pericentromeric chromatin structure.

### Polymer model predicts changes in chromatin dynamics at pericentromeres as observed *in vivo*

**Figure 4:**
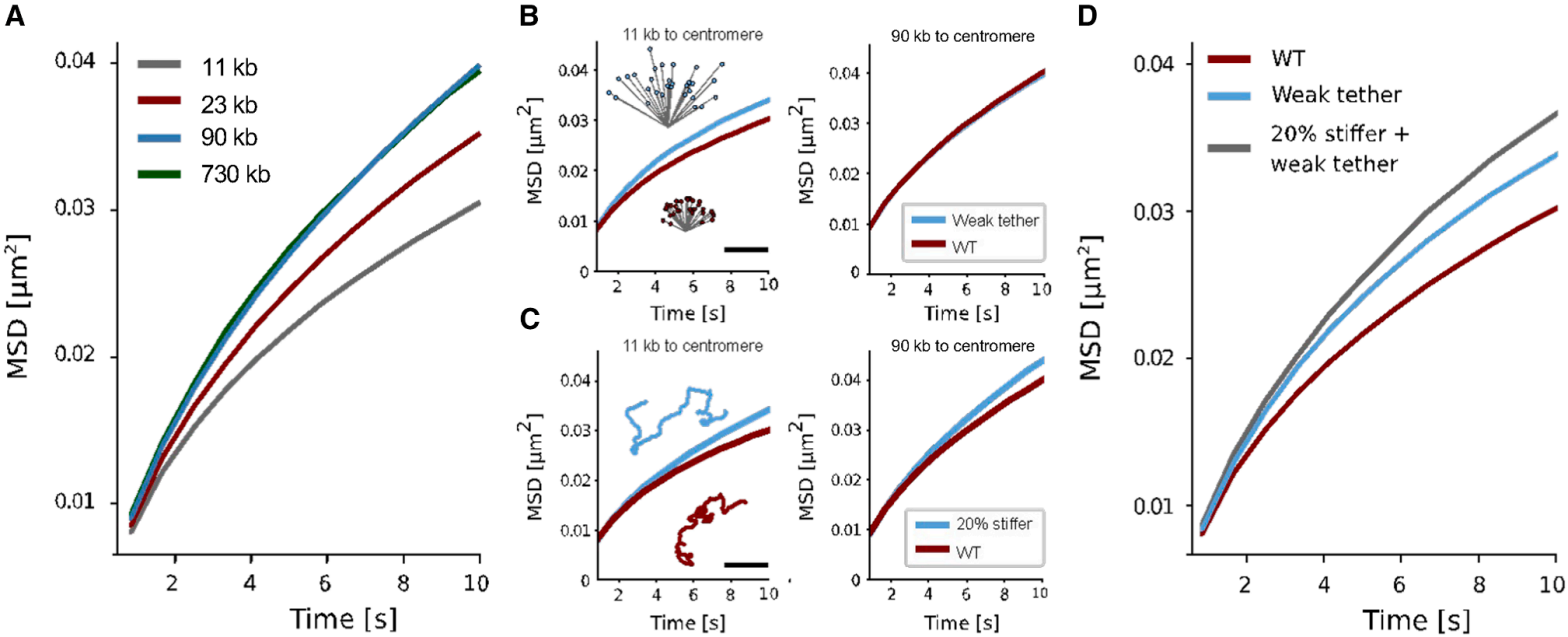
Polymer model recapitulates changes in chromatin dynamics at pericentromeres (A) Comparison of MSDs for loci with various distances to the centromere. (B) Effect of increasing the flexibility of the SPB tether from rigid (red curves) to a spring constant of 1kbT/(105 nm)^2^, which would allow for a standard deviation of 74 nm (105 nm/sqrt(2)) if no other forces were applied (blue curves). Left plot shows loci 11 kb from the centromere, and the right shows loci 90 kb from the centromere. Only loci on chromosomes larger than 400 kb, excluding Chr XII, were included. The inset shows a 2D slice of the centromere positions for the loose (blue) and rigid (red) centromeres. Bonds are shown in gray and scale bars are 500 nm. (C) Effect of increasing polymer stiffness from one leading to a persistence length of 70 nm (red curves) to one with an angular spring constant that is 20% higher (blue curves). Loci are as in (B). Inset shows a 2D conformation of Chr I in the two conditions; scale bars are 500 nm. (D) MSD for loci 11 kb away from the centromere when comparing effects of increasing flexibility (blue and red curves, same as left panel of (B)) and combining flexibility and stiffness (gray curve).

We next used biophysical polymer simulations (Materials and methods) to determine if Smc5 binding to microtubules and chromatin structure could together drive chromatin dynamics in the pericentromeric region. Initially, we assessed chromatin mobility at positions corresponding to 11, 23, 90 and 730 kb from the centromere of different chromosomes. Polymer simulation showed that the increase in MSD_10s_ was proportional to the distance to the centromere, as observed *in vivo,* and plateaued at 90 kb (Figure 4A compared to Figure 1C). Although the increase in mobility in the polymer model was less pronounced than in the experimental data, the relative characteristics were maintained, thus validating the use of the polymer to investigate the effects of perturbations.

We then altered parameters related to microtubule tethering and chromatin stiffness and analyzed the resulting changes in simulated chromatin dynamics. Firstly, we examined the effect of weakening the tethering of microtubules to the kinetochore. To this end, the spring constant of the harmonic bond between the centromere and the SPB was reduced from a value ensuring a fixed bond to one yielding an energy of 1kbT at 105 nm extension from the 400 nm rest length (Figure 4B). Loosening the attachment increased mobility of loci located 11 kb away from the centromere, as we observed when microtubules were depolymerized by nocodazole, but did not alter loci located 90 kb away (Figures 2D, 4B, and S7), indicating that microtubule tethering acts locally on chromatin mobility (see discussion). Secondly, we modified the chromatin state by increasing its global stiffness. In contrast to the position-dependent change observed when microtubule tethering was weakened, this resulted in an increase in global mobility, independent of the distance from the centromere (Figures 4C and S8), in agreement with previous observations.^38^ These results altogether suggest that pericentromeric chromatin dynamics are locally constrained by microtubule attachments at the centromere and globally modulated by chromatin stiffness.

We reasoned that both effects could be at play in the pericentromeric region since the Smc5-2KE mutation displayed a greater increase in mobility than the nocodazole treatment. To test this hypothesis, we modified the polymer model parameters to associate an increase in chromatin stiffness with a relaxation of chromatin tethering to microtubules. We observed that increasing chromatin stiffening further enhanced chromatin dynamics, reflecting the *in vivo* differences observed between WT cells treated with nocodazole and the *smc5-2KE* mutant (Figures 2D, 4D, and S9). These results show that weakened microtubule attachment combined with stiffer chromatin is sufficient to account for the increase in pericentromeric chromatin dynamics observed *in vivo* in the *smc5-2KE* mutant. They also reinforce our model that Smc5 binds to both microtubules and chromatin to maintain proper pericentromeric chromatin folding and dynamics.

### The *smc5-2KE* mutant alters mitotic spindle integrity

**Figure 5:**
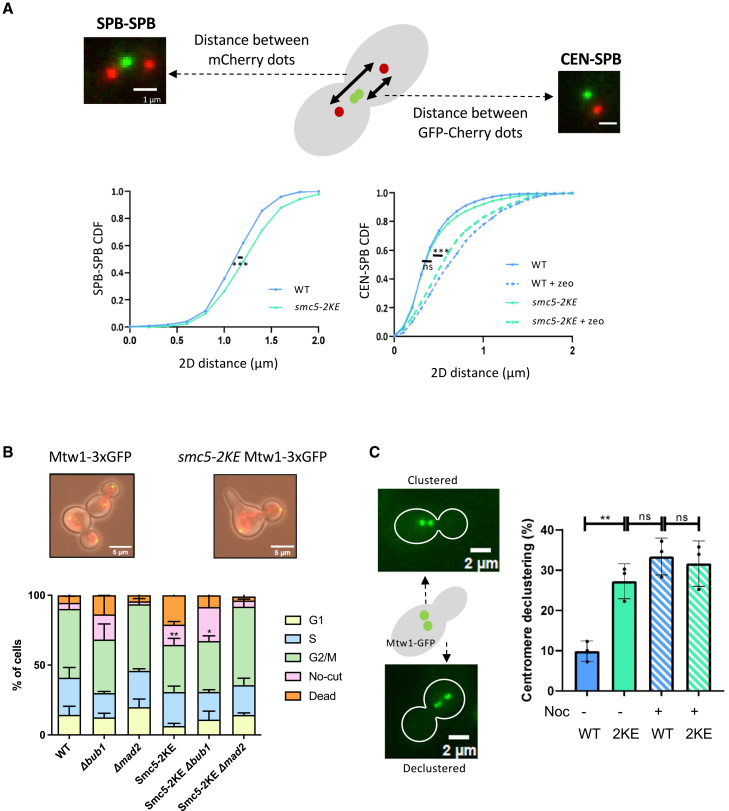
The *smc5-2KE* mutant alters the mitotic spindle integrity (A) CEN IV was tagged with GFP and SPB with Spc42-mCherry. Representative images are shown. Scale bars are 1 μm. 2D distances between two SPBs (red dots) (left) and between CEN (green) and SPB (red) in G2/M cells (right) were measured in three independent biological replicates. Number of cells for SPB-SPB (N_WT_ = 224, N_2KE_ = 332) and for CEN-SPB (N_WT_ = 247, N_2KE_ = 380). As a positive control, CEN-SPB was measured in the presence of Zeocin with N_WT ZEO_ = 1299, N_2KE ZEO_ = 2033. Statistical significance was assessed using a two-sided Wilcoxon rank-sum test; ns, not significant; ∗∗∗*p* < 0.0001. (B) Examples of cycling and defective mitotic cells are shown (top) and cycle phase distribution quantification (bottom) in *Mtw1-3xGFP* (WT) and *Smc5-2KE Mtw1-3xGFP* (2 KE) cells, in the presence or absence of deletion of *MAD2 (Δmad2*) or *BUB1* (*Δbub1*). Scale bars are 6 μm. Cells were categorized into G1, S, or G2/M phases. Additionally, dead cells and those exhibiting no-cut phenotype (no-cut) were counted in three experimental replicates N number of cells were counted N_WT_ = 439, N_WT NOC_ = 588, N_2KE_ = 413, N_2KE NOC_ = 652. Statistical significance was assessed using an unpaired two-tailed *t* test; ∗∗*p* < 0.01 and ∗∗∗*p* < 0.001. (C) Mtw1 was labeled with 3xGFP to measure kinetochore clusters with or without nocodazole addition (15 ng/μL). Representative images are shown. Scale bars are 2 μm. Centromere clustering was quantified by counting the number of Mtw1-3xGFP foci per cell in at least 100 cells per condition. Cells were classified as exhibiting either a single focus (clustered) or multiple foci (declustered). The percentage of cells with fragmented foci was calculated and compared between WT and *smc5-2KE* mutant. Statistical significance was assessed using an unpaired two-tailed *t* test; ns, not significant; ∗∗∗*p* < 0.001.

Since a correlation between pericentromeric chromatin compaction and spindle elongation has been reported previously, both in yeast and mammals,^10^^,^^59^^,^^60^ we sought to determine whether the Smc5-2KE mutation could alter spindle elongation. We studied spindle length by measuring the distance between each SPB in dividing G2/M cells. We used SPB protein Spc42 fused to mCherry^61^ to calculate the 2D distances between the two fluorescent centers of mass as above (Figures 3 and 5A left). In WT cells, spindle length average was 1.18 ± 0.29 μm, whereas the *smc5-2KE* mutant exhibited a relatively small but significant increase to 1.32 ± 0.33 μm (*p* value <0,0001) (Figure 5A left), suggesting that the binding of Smc5 to microtubules contributes to the regulation of spindle length.

The length of the spindle may change due to variations in the length of the kinetochore microtubules connecting centromeres to the SPB, or to modifications in the kinetochore/pericentromere itself.^62^ Measuring the distance between GFP-tagged CEN IV and Spc42-mCherry showed no difference between the WT and *smc5-2KE* mutant (Figure 5A right), while Zeocin control increased CEN-SPB distance as already demonstrated (Figure 5A right).^38^^,^^58^ Within the resolution of this assay, *smc5-2KE* does not cause a major change in kinetochore microtubule length, indicating that the *smc5-2KE* mutation does not disrupt the basic attachment of kinetochores to microtubules. To also evaluate the kinetochore integrity in the *smc5-2KE* mutant, we measured centromere clustering *in vivo* by visualizing kinetochore protein Mtw1 fused to 3xGFP at the endogenous locus.^8^ Mtw1 is a kinetochore-specific protein, part of the MIND complex, which links the inner and outer layers of the kinetochore.^63^ While Mtw1-3xGFP-expressing cells cycled normally, combination with *smc5-2KE* led to an unexpected mitosis defect reminiscent of the no-cut phenotype, where completion of cytokinesis is delayed (Figure 5B right).^64^ Strikingly, deletion of *MAD2* or *BUB1*, key spindle assembly checkpoint (SAC) components, suppressed the observed mitotic defect, allowing cells to progress through mitosis despite the presence of the *Smc5-2KE* mutation and *Mtw1-3xGFP* (Figure 5B right).

In WT cells, Mtw1-3xGFP formed a single fluorescent focus due to the centromeric clustering typical of the Rabl configuration (Figure 5C, up, Figure S10). Treatment with the microtubule-depolymerizing drug nocodazole induced fragmentation of the Mtw1-3xGFP labeling into several foci, consistent with centromere declustering, as previously reported (Figure 5C, bottom, Figure S10).^8^^,^^65^ Centromere clustering was quantified by counting Mtw1-3xGFP foci per nucleus: nuclei with a single focus were classified as “clustered,” whereas nuclei with more than one focus were classified as “declustered.” It is striking to note that the *smc5-2KE* mutation alone is sufficient to cause centromere disassembly in the absence of nocodazole to a similar extent as that associated with the addition of nocodazole in the WT strain (27.26 ± 4.3% and 9.89 ± 2.5% for *smc5-2KE* and WT cells, respectively, Figures 5C and S10).

In conclusion, the Smc5-2KE mutation, combined with the Mtw1-3xGFP hypomorphic allele, exacerbates a kinetochore defect that activates the SAC. This suggests that the molecular link between chromatin and microtubules provided by Smc5/6 participates in kinetochore maintenance and mitotic spindle integrity.

### The *smc5-2KE* mutant affects homologous recombination at pericentromeres

**Figure 6:**
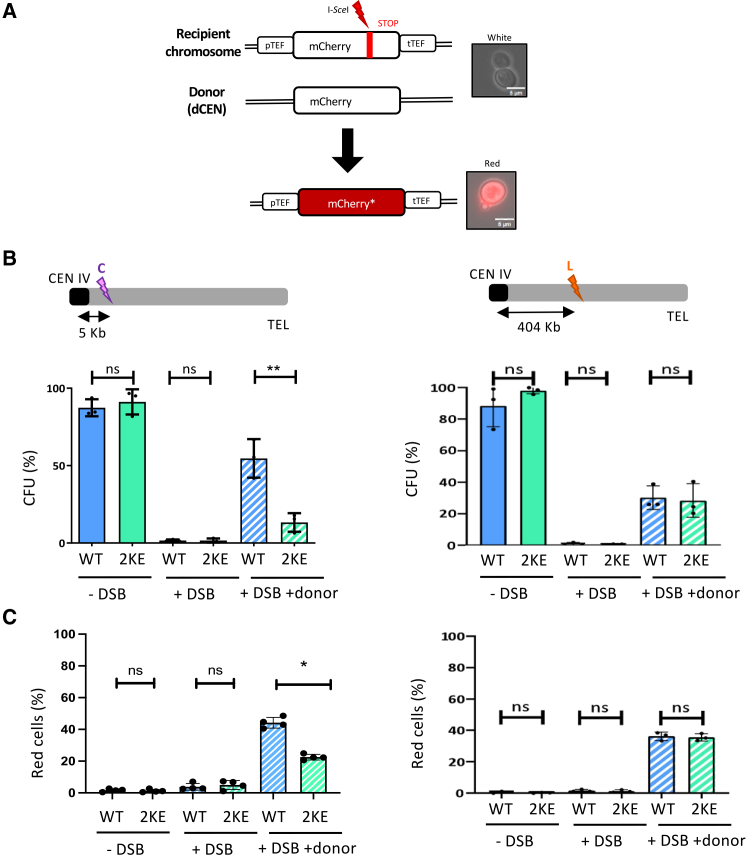
The *smc5-2KE* mutant decreases homologous recombination efficiency at pericentromeres (A) Schematic representation of the system to track homologous recombination (HR). In the recipient mCherry, I-*Sce*I has a 30 bp I-*Sce*I cutting site sequence followed by a stop codon (red bar) inserted at 57 amino acids from the end of the mCherry coding sequence. The donor (dCen) is a full-length mCherry lacking promoter and terminator sequences, carried on a centromeric plasmid. A functional mCherry could be expressed only after successful HR. Representative images are shown. Scale bars are 6 μm. (B) Colony formation unit (CFU) test showing three conditions: without DSB (-DSB), with a DSB (+DSB), and with a DSB and a donor (DSB+donor) in the C and in the L strain for the WT and for the *smc5-2KE* mutant. Ratio between induced I-*Sce*I/non induced I-*Sce*I. Three experiments were conducted with the number of cells counted as follows: For the C strain, N_WT PRS_ = 487, N_WT PB_ = 528, N_WT Cen_ = 668, N_2KE PRS_ = 330, N_2KE PB_ = 620, N_2KE Cen_ = 752. For the L strain, N_WT PRS_ = 344, N_WT PB_ = 309, N_WT Cen_ = 328, N_2KE PRS_ = 261, N_2KE PB_ = 379, N_2KE Cen_ = 387. Statistical significance was assessed using a two-sided Mann–Whitney test; ns, not significant; ∗∗*p* < 0.01. (C) Number of red cells (%) without DSB (-DSB), with a DSB (+DSB), and with a DSB and a donor (DSB+donor) in the C and in the L strain. Three experiments were conducted, counting the number of cells for the C strain as follows: N_WT PRS_ = 707, N_WT PB_ = 719, N_WT Cen_ = 2180, N_2KE PRS_ = 727, N_2KE PB_ = 660, N_2KE Cen_ = 2553. For the L strain, the counts were N_WT PRS_ = 506, N_WT PB_ = 607, N_WT Cen_ = 1299, N_2KE PRS_ = 744, N_2KE PB_ = 712, N_2KE Cen_ = 1551. Statistical significance was assessed using a two-sided Mann–Whitney test; ns, not significant; ∗*p* < 0.05.

Our results above indicate that the integrity of the mitotic spindle, which relies on both microtubules and pericentromeric chromatin, requires Smc5 interaction with microtubules. We therefore examined whether pericentromeric chromatin remains robust upon DNA damage such as DSBs. We induced a single site-specific DSB either in the pericentromere or at a distal chromosome location. We then compared the repair efficiency of these breaks by HR in both WT and *smc5-2KE* mutant strains according to^35^^,^^66^ (Figure 6A). To this end, I-*Sce*I was expressed on a plasmid with the galactose-inducible *GAL1-10* promoter to induce the DSB (+DSB), while an empty vector served as a control (-DSB). In this assay, successful HR in the presence of a donor leads to mCherry expression, detectable by red fluorescence in living cells (Figure 6A). The mCherry recipient sequence containing an I-*Sce*I target site was introduced at 5 kb (named C) and 404 kb (named L) from CEN IV in WT and *smc5-2KE* mutant strains (Figure 6B). The I-*Sce*I cleavage efficiency in these strains, quantified by qPCR, was ∼80%, after 6 h of I-*Sce*I induction, irrespective of the position of the cutting site, as previously observed^66^ (Figure S11A). Cell survival was assessed by colony-forming unit (CFU) assays. As expected, in the absence of I-*Sce*I cut, WT and *smc5-2 kE* C and L colonies grew in both inducing and non-inducing conditions (Figure 6B).

Interestingly, providing the mCherry donor sequence on the dCEN plasmid to allow DSB repair by HR had different outcomes in *smc5-2KE* depending on the DSB position. When the DSB was in the pericentromeric region, the *smc5-2KE* mutant showed significantly reduced survival rates compared to the WT (C strains, 15 ± 6% and 55 ± 12.4%, respectively, Figure 6B). In contrast, both WT and the *smc5-2KE* mutant presented similar survival rates for a DSB located in the L position (40 ± 7.5% for both L strains) (Figure 6B). In addition, when the DSB was pericentromeric, the *smc5-2KE* mutant generated fewer mCherry-positive cells among survivors than the WT 44 ± 3.4% and *smc5-2KE* 22.5 ± 1.7% of red cells). This discrepancy was not observed when the DSB was at the L position (36 ± 2.5% of red cells for both). We confirmed that the HR defect was not due to loss of the centromeric plasmid, as plasmid maintenance efficiency of pRS415 was comparable between WT and smc5-2KE cells under our experimental conditions (Figure S11B), nor was there altered Smc5/6 recruitment at DSB, as ChIP-qPCR of Smc5-MYC or Smc5-2KE-MYC revealed no significant differences in their occupancy, irrespective of the DSB position (Figures S11C and S11D).

Together, these findings indicate that *smc5-2KE* specifically compromises HR at pericentromeres, while leaving HR along the chromosome arm largely unaffected. They further support a model in which Smc5 binding to microtubules drives pericentromeric folding, a condition essential for maintaining pericentromeric integrity.

## Discussion

SMC complexes share a fundamental role in chromatin loop formation, and their enrichment at centromeres and/or adjacent regions of chromosomes is an evolutionarily conserved feature of eukaryotic cells.^17^^,^^67^^,^^68^ Polymer-physics models predict that SMCs-induced chromatin loops at pericentromeres generate a structure described as a bottlebrush, which balances the outward pulling forces of microtubules with the inward attractive forces of chromatin during cell division.^4^ Smc5/6 associates with microtubules in budding yeast and *Xenopus*^19^^,^^20^^,^^69^ (our present data - Figure S1). However, the role of this association in organizing pericentromeric structure *in vivo* was previously unknown. Based on structural studies, we generated a mutant that specifically decreases the affinity of Smc5 to microtubules. Consequently, chromatin mobility along the chromosome is modified, as well as chromatin properties (decompaction and/or stiffness) in the pericentromeric region. Smc5/6 binding to microtubules specifically maintains the integrity of the mitotic spindle and contributes to DSB repair efficiency in the pericentromeric region.

We provide evidence that Smc5/6 offers a molecular link to microtubules through the conserved residues K624 and K631 in the hinge region of Smc5. First, alphafold3 modeling shows a high probability of interaction between the extruded Smc5 hinge and alpha/beta dimers of tubulin (Figures 1A and 1B). Second, the microtubule binding co-sedimentation assay with Smc5 is decreased when K624 and K631 are mutated to aspartic acid (Figure 1C). Third, in the *smc5-2KE* mutant, chromatin dynamics mirrors microtubule depolymerization (Figure 2D) and mimics the effects observed in the *ndc80-1* kinetochore mutant (Figure 2E). The ability to bind microtubules is a novel and important function of the Smc5/6 complex.

The association between Smc5/6 and microtubules restricts chromatin mobility and has critical consequences for the folding of the pericentromeric region. Measurements of chromatin mobility and intra-chromosomal distances in the *smc5-2KE* mutant indicate that reduced association of Smc5/6 with microtubules affects pericentromeric chromatin structure. Biophysical polymer simulations support a model whereby Smc5/6 binding to microtubules would lead to increased chromatin rigidity or decompaction (Figure 4D). Notably, changes in the nuclear environment are consistent with increased chromatin mobility with a similar alpha exponent of the MSD (Figure 1E). How could the Smc5-2KE mutation affect the pericentromeric chromatin? FRAP experiments have revealed that loss of microtubule polymerization or kinetochore mutants results in decreased levels of histone H4 and H2B in the pericentromere.^62^ Because histone reduction has been linked to chromatin decompaction and increased chromatin dynamics,^7^^,^^46^^,^^58^ disruption of microtubule-induced tension in the *smc5-2KE* mutant could similarly result in decreased histone occupancy, chromatin decompaction, and subsequent enhanced chromatin mobility. Since microtubule loss also augments intra-chromosomal contacts and affects pericentromeric configuration,^11^ the tension created by microtubules could also regulate chromatin structure near attachment sites.^11^ Accordingly, the interaction between Smc5 and microtubules, disrupted in the *smc5-2KE* mutant, may contribute to microtubule-induced tension and spindle robustness, thereby influencing pericentromeric chromatin architecture.

Alternatively, Smc5/6 might regulate pericentromeric chromatin structure via its DNA compaction activity, as recently shown by single-molecule experiments with purified human and yeast Smc5/6.^20^^,^^70^ Mechanistically, the altered chromatin structure in the *smc5-2KE* mutant could result from impaired loop extrusion by Smc5/6 dimers.^17^ However, we show here that acute depletion of the entire complex - thereby abolishing loop extrusion activity - has an impact on chromatin mobility opposite to the *Smc5-2KE* mutation (Figure S6). This finding strongly suggests that loop extrusion is not responsible for the altered chromatin organization in *smc5-2KE.* Instead, Smc5/6 could promote chromatin compaction through alternative mechanisms, such as microtubule-associated nucleosome stabilization, a hypothesis to be tested in future work. Defects in cohesin or condensin recruitment could also contribute to pericentromeric decompaction or stiffness, leading to increased chromatin dynamics (Figure 1C).^6^^,^^45^^,^^46^ Pairing defects by cohesin appear unlikely in the *smc5-2KE* mutant, as cohesion is intact, contrasting what is observed in Smc1 cohesin-depleted cells (Figures S6D and S6E). Overall, our observations suggest a model in which microtubule forces transmitted via Smc5/6 regulate pericentromeric chromatin folding and stiffness, independently of loop extrusion or cohesion.

It is striking that disrupting the microtubule binding to centromeres, either by depolymerizing microtubules with nocodazole, altering the kinetochore in the *ndc80-1* mutant, or using the *smc5-2KE* mutant, results in increased chromatin dynamics not only close to the centromere but also, to a lesser extent, at more distant genomic regions. It is possible that once centromeres are freed from microtubules, they exhibit increased movement, which could be translated into greater chromatin mobility further along the chromosome. By analogy, a rope with movement at one end could lead to increased movement at the other end. However, by simulating weakened microtubule anchoring, the polymer model fails to fully reproduce the *in vivo* increase in chromatin dynamics away from the centromere (Figure 4B), possibly due to unmodeled features such as SMC-mediated loop formation or centromere declustering, which could influence chromatin density around pericentromeric domains *in vivo* due to a modified Rabl configuration.

Smc5-2KE specifically affects the mitotic spindle in a SAC-dependent manner when the SAC proteins *MAD2* or *BUB1* are deleted. SAC is activated when kinetochores are improperly attached, and we indeed observed that the SAC is activated when the *smc5-2KE* mutant is combined with Mtw1-3xGFP, consistent with exacerbated kinetochore dysfunction in this background. Furthermore, in the same combination, we also observed a defect in cytokinesis (Figure 5B) similar to the defect, named “no-cut,” previously reported in mutant cells in kinetochore proteins, *ndc10-1* and *ndc80-1* alleles,^64^^,^^71^ and in cells with incomplete DNA segregation.^72^ We propose that combining the Smc5-2KE mutation with Mtw1-3xGFP compromises kinetochore structure,^73^ causing chromosome segregation defects severe enough to induce the “no-cut” phenotype. This highlights the pivotal role of Smc5/6 in organizing pericentromeric chromatin and kinetochores, through its association with microtubules.

In budding yeast and mammalian cells, Smc5/6 is enriched around DSBs^26^^,^^27^^,^^74^ and contributes to HR by resolving Holliday junctions,^22^^,^^30^^,^^31^ as well as by maintaining DSB ends together.^28^ In line with the role of Smc5/6 in HR, we show that the Smc5-2KE mutation reduces repair efficiency, specifically at pericentromeres (Figure 6). HR efficiency may be reduced in the *smc5-2KE* mutant either because pericentromeric chromatin properties are altered, consistent with our observation that Smc5/6 fails to interact with microtubules (Figure 3), or because the *smc5-2KE* mutant directly impairs pericentromeric DSB repair. For instance, the mutation of the hinge domain of Smc5, which has an affinity for ssDNA *in vitro,*^34^^,^^75^ may affect this interaction at DSBs *in vivo*, and consequently impact 5′ DNA resection at DSBs or the maintenance of the DSB ends.^28^ Alternatively, the loss of association between microtubules and Smc5/6 in the mutant may hinder the efficient relocalization of damaged pericentromeres to the nuclear periphery. This is consistent with previous findings showing that Smc5/6 relocalization is required for the repair of DSBs in repetitive rDNA sequences in budding yeast^24^ and in heterochromatin in *Drosophila*.^76^^,^^77^ Furthermore, 5′ DNA resection at pericentromeric DSBs may lead to detachment of the centromere from microtubules, triggering the formation of damage-induced microtubules (DIMs). Capture of the centromere freed from the microtubules, as previously proposed,^78^ would require Smc5/6. A mutation affecting association with DIMs could hamper centromere recapture and efficient DSB repair.

Smc5/6 is involved in multiple cellular processes, yet the specific reduction in microtubule binding affinity observed in the *smc5-2KE* mutant—without broad disruption of other Smc5/6 functions—suggests that this interaction may play a direct role in the observed chromatin dynamics defects. The conservation of the hinge domain lysines (K624 and K631) and their critical role in microtubule interaction further support this model. However, we cannot exclude the possibility that cumulative defects in other Smc5/6 functions contribute to the phenotype. Future studies will be essential to dissect the relative contributions of microtubule binding versus other Smc5/6 activities in maintaining pericentromeric chromatin integrity.

**Figure 7:**
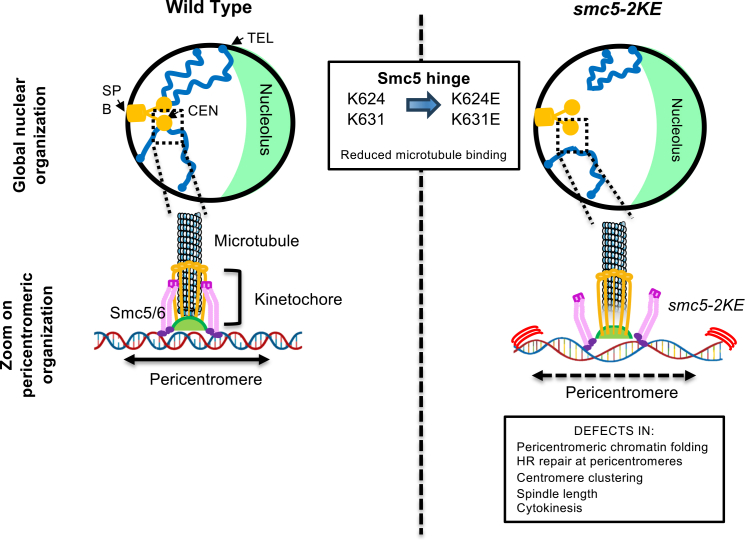
Working model for Smc5/6 coupling to microtubules in chromatin dynamics and pericentromeric organization On the top, schematic global views of two yeast chromosomes with centromeres (CEN) linked to nuclear microtubules emanating from the spindle pole body (SPB) and telomeres (TEL) attached to the nuclear envelope. On the bottom a zoom of the microtubule/pericentromeric organization. (left) In wild-type cells on the left, Smc5/6 (pink) binds to microtubules (blue cylinder), constraining the dynamics of pericentromeric chromatin and maintaining proper organization around the centromere. The kinetochore (green) is properly attached to microtubules. (right) In the *smc5-2KE* mutant, Smc5-microtubule interaction is weakened, and pericentromeric chromatin becomes more dynamic (red waves) and less constrained. This, in turn, sensitizes the cell to defects in kinetochore function, leading to spindle and cytokinesis problems, spindle checkpoint activation, and a decrease in homologous recombination repair at pericentromeres.

In conclusion, focusing on chromatin dynamics has proven to be a powerful approach for understanding the functional relevance of the Smc5/6—microtubule interaction in maintaining pericentromeric chromatin integrity. We propose that the enrichment of Smc5/6 at pericentromeres, together with its coupling to microtubules, protects pericentromeric chromatin integrity against mitotic forces (Figure 7). Smc5/6 enrichment in the pericentromeric region is conserved throughout mitosis of many organisms.^15^^,^^27^^,^^79^ While structural predictions for the human Smc5/6-tubulin interaction are not yet available, the conservation of critical lysine residues (K624 and K631) in the Smc5 hinge domain, combined with the high sequence homology of tubulin (∼87%), suggests a conserved interaction interface. This conservation, along with the functional enrichment of Smc5/6 at pericentromeres during mitosis, makes it likely that a similar association between Smc5/6 and microtubules occurs in mammals during open mitosis, the only phase when microtubules interact with centromeric regions in mammals. Therefore, future studies in human cells will be essential to explore the role of Smc5/6 in preserving pericentromeric structure during mitosis and its broader implications for mitotic regulation and genome stability.

### Limitations of the study

While the consistent differences between WT and *smc5-2KE* cells across matched conditions support our main conclusions and indicate a role for microtubule-associated Smc5/6 in chromatin dynamics regulation, direct and indirect effects remain difficult to fully disentangle. The TetO-TetR-GFP arrays used for locus tracking may disrupt local chromatin architecture and dynamics, particularly in pericentromeric regions. Additionally, nocodazole could influence chromatin behavior through mechanisms beyond microtubule depolymerization. Finally, the *smc5-2KE* allele only partially impairs Smc5-microtubule binding. Further in-depth studies will be required to fully elucidate how the SMC5/6 complex associates with microtubules.

## Resource availability

### Lead contact

Further information and requests for resources and reagents should be directed to and will be fulfilled by the lead contact, Emmanuelle Fabre emmanuelle-g.fabre@inserm.fr.

### Materials availability

All yeast strains, plasmids, and custom oligonucleotides generated in this study are available from the lead contact upon reasonable request, subject to material availability.

### Data and code availability

• **Data:** The datasets generated during this study have been deposited in Zenodo and are publicly available as of the date of publication under Zenodo: 18208813. Mass spectrometry datasets are deposited in MassIVE:MSV000101977. All identifiers are listed in the key resources table.
• **Code:** Custom scripts used for polymer simulations are deposited in Zenodo: 13754654.
• **Other items:** Any additional information required to reanalyze the data reported in this paper is available from the lead contact upon request.

## Acknowledgments

We thank A. Marston, P. Hieter, and T. Teixeira for sharing strains and plasmids. We also thank E. di Santo and C. Marquez for their participation in strains constructions. The authors thank the OHRI Proteomics Core at the Ottawa Hospital Research Institute for performing the mass spectrometry experiments and proteomic data analysis for this study. Our acknowledgments go to S. Grosse-Holz, X. Zhao, and J-M. Victor for enlightening discussions, P. Lesage, F. Garcia-Fernandez, and S. Mihic for their constructive reading of the manuscript. P. Therizols and the members of the laboratory are thanked for the many lively discussions on this project, and the GDR Architecture Dynamique du noyau et des génomes (ADN&G) for the valuable exchanges with its community. This work was supported by an FRM grant to EF (EQU202203014635) and a CIHR grant to DD (FDN–167265). EF is also supported by PLBIO-INCA (PLBIO20-312) and DD by a Canada Research Chair in Chromatin Dynamics & Genome Architecture (CRC-2023-00405). ACS was supported by FRM (ECO202006011576) and ARC (ARCDOC42023010006078) fellowships.

## Author contributions

A.C.S. performed the experiments in Figures 1A, 1B, 1E, 2, 3, 5, 6, S2E, S2G–S2I, S3, S4, S5, S6, S10, and S11. C.M. performed the experiments in Figure 6. S.I. performed the experiments in Figures 1D, S1, S2A, S2C, S2D, S2F and constructed the strains for Figure 2E. R.B. performed the experiments in Figures 5A, 6, and S6. T.F. performed the experiments in Figure 1F. H.D.P. performed the experiments in Figures 4, S7, S8, S9. G.L. performed the experiments in Figure 1C and S2B. J.R. performed alpha-fold modeling in Figures 1B and S1D. A.C.S. and E.F. wrote the manuscript with review and editing by M.B., L.M., and D.A.

## Declaration of interests

The authors declare no competing interests.

## STAR★Methods

### Key resources table

**Table:** 

REAGENT or RESOURCE	SOURCE	IDENTIFIER
**Antibodies**		

Mouse anti-MYC antibody Chip grade (9E10-sc40)	Santa Cruz Biotechnology	AB_627268
Mouse monoclonal anti-HA	Sigma	AB_262051
Rabbit polyclonal anti-yH2A	Abcam	AB_301630
Mouse anti-Strep antibody	Qiagen	AB_2810987
Mouse anti-Myc (9E10)	GeneTex	AB_368676

**Chemicals, peptides, and recombinant proteins**		

Nocodazole	Sigma	M1404
Hydroxyurea	Sigma	H8627
Camptothecin	Sigma	C9911
4-Nitroquinoline 1-oxide	Sigma	N8141
Alpha-factor	Zymo Research	Y1001M
RNase A	Roche	10109169001
SYTOX Green	Sigma	S7020

**Deposited data**		

Datasets generated during this study	Zenodo: 18208813	N/A
Custom scripts used for polymer simulations	Zenodo: 13754654	N/A
Datasets from Mass spectrometry, accession code MSV000101977	MassIVE: MSV000101977	N/A

**Experimental models: Organisms/strains**		

*S. cerevisiae*: Strain background: BY4741	N/A	N/A
See Table 1 for strains	N/A	N/A

**Oligonucleotides**		

See Table S1 for oligonucleotides	N/A	N/A

**Recombinant DNA**		

Cas9 and a gRNA scaffold	Arbona et al.^80^	N/A
gRNA to cut close to the SMC5-K631. – KanMX2	This study	N/A
pFA6a-13MYC-URA3	P. Lesage	N/A
pTEF-tTEF-LEU2-LacO	Therizols et al.^80^^,^^81^	N/A
LacI-GFP-HIS3	N/A	N/A
AFB2-URA3	T. Teixeira	N/A
gRNA to cut close to the SMC5-K631 -HIS3	This study	pJH2970
I*-Sc*eI-GAL1-10	García-Fernandez et al.^66^	pB07
mCherry plasmid	García-Fernandez et al.^66^	N/A

**Software and algorithms**		

Fiji (ImageJ)	Schindelin et al.^82^	https://imagej.net/software/fiji
MATLAB	MathWorks	https://www.mathworks.com/products/matlab.html
Polychrom	Open2C	https://github.com/open2c/polychrom
OpenMM	Eastman et al.^83^	http://openmm.org
AlphaFold3	Abramson et al.^84^	https://alphafoldserver.com/
GraphPad Prism version 11.0.1 for MacOS	GraphPad Software, Boston, Massachusetts USA,	https://www.graphpad.com

**Other**		

Attune NxT Flow Cytometer	Thermo Fisher Scientific	N/A
Nikon Ti-E widefield microscope	Nikon	Plan APO
Andor *Neo* sCMOS camera	Oxford Instruments/Andor	N/A
QuantStudio 5 Real-Time PCR System	Thermo Fisher Scientific	N/A
epMotion 5070 robot	Eppendorf	N/A
MagNA Lyser	Roche	N/A
Bioruptor Pico sonicator	Diagenode	N/A

### Experimental model and study participant details

#### Yeast strains

All Saccharomyces cerevisiae strains used in this study are listed in Table S1 and are isogenic to the BY4741 background.

**Table 1:** Genotypes of used strains

Common name	Strain	Genotype	Reference
11 kb Chr IV	YPE4019	Mata, ura3, trp1-1, leu2-3, his3-11,15, can1-100, ade2-1, P_MET3_-CDC20::URA3, Spc42-tdTomato::NATMX, leu2:: P_URA3_-TetR-GFP::LEU2, tetOx224-URA3 (tetOs 11,5 kb to right of CENIV), yil015w::KANMX	Paldi et al.^11^
11 kb-2KE Chr IV	YPE4144	Mata, ura3, trp1-1, leu2-3, his3-11,15, can1-100, ade2-1, P_MET3_-CDC20::URA3, Spc42-tdTomato::NATMX, leu2:: P_URA3_-TetR -GFP::LEU2, tetOx224-URA3 (tetOs 11,5 kb to right of CENIV), yil015w::KANMX, YOL034W::K624E, YOL034W::K631E	This study
23 kb Chr IV	YEF1659	Mata, ura3, trp1-1, leu2-3, his3-11,15, can1-100, ade2-1, P_MET3_-CDC20::URA3, Spc42-tdTomato::NATMX, leu2:: P_URA3_-TetR -GFP::LEU2, tetOx224-URA3 (tetOs 11,5 kb to right of CENIV)	Paldi et al.^11^
23 kb-2KE Chr IV	YPE4145	Mata, ura3, trp1-1, leu2-3, his3-11,15, can1-100, ade2-1, P_MET3_-CDC20, Spc42-tdTomato::NATMX, leu2:: P_URA3_-TetR -GFP::LEU2, tetOx224-URA3 (tetOs 23 kb to right of CENIV), YOL034W::K624E, YOL034W::K631E	This study
90 kb Chr IV	YEF1081	Mat alpha, ura3Δ0 or-851, his3Δ1, can1::HPHMX, his3::HIS3-LacI-GFP, YDR042C::lacO-LEU2	This study
90 kb-2KE Chr IV	YEF1654	Mat alpha, ura3Δ0 or-851, his3Δ1, can1::HPHMX, his3::HIS3-LacI-GFP YDR042C::lacO-LEU2, YOL034W::K624E, YOL034W::K631E	This study
730 kb Chr IV	YEF1068	Mat alpha, ura3Δ0, leu2Δ0, his3Δ1, lys2Δ202, ade2-661, iyGL117::TetR-mRFP-NATMX, YDR354W::tetO-URA3 HIS3::HIS3-LacI-GFP YDR259C::lacO-LEU2	This study
730 kb-2KE Chr IV	YEF1706	Mat alpha, ura3Δ0, leu2Δ0, his3Δ1lys2Δ202, ade2-661, iyGL117::tetR-mRFP NATMX YDR354W::tetO-URA3 HIS3::HIS3-LacI-GFP, YDR259C::lacO- LEU2, YOL034W::K624E, YOL034W::K631E	This study
90 kb-2KR Chr IV	YPE4180	Mat alpha, ura3Δ0, trp1Δ63, leu2Δ0, his3Δ1, lys2Δ202, can1::HPHMX, ade2-661, lys2::TetR-GFP-LYS2, yel053c::KanMX::tetO-URA3, Chr IV 453880 + 453959::KanMX:: mCherry∗I*Sce*I(cs), YOL034W::K624R, YOL034W::K631R	This study
90 kb Chr V	YEF1454	Mat alpha, ura3Δ0, trp1Δ63, leu2Δ0, his3Δ1, lys2Δ202, can1::HPHMX, ade2-661, lys2::TetR-GFP-LYS2, yel053c::KanMX:: tetO-URA3, Chr IV 453880 + 453959::KanMX:: mCherry∗I*Sce*I(cs)	García-Fernandez et al.^48^
90 kb-2KE Chr V	YEF1666	Mat alpha, ura3Δ0, trp1Δ63, leu2Δ0, his3Δ1, lys2Δ202, can1::HPHMX, ade2-661,::TetR-GFP-LYS2, yel053c::KanMX:: tetO-URA3, Chr IV 453880 + 453959:: KanMX:: mCherry∗I*Sce*I(cs), YOL034W::K624E, YOL034W::K631E	This study
SMC6-AID AFB2	YPE4059	Mat a, trp1-1, leu2-3, his3-11, ade2-1, P_MET3_-CDC20::URA3, Spc42-tdTomato::NATMX, leu2::PURA3::tetR-GFP::LEU2, tetOx224-URA3 (tetOs 11,5 kb to right of CENIV), Smc5-AID-9myc-KANMX, his3-11,15:: pEF604 (=AFB2-HIS3)	This study
SMC1-AID AFB2	YPE4173	Mat a, trp1-1, leu2-3, his3-11, ade2-1, P_MET3_-CDC20::URA3, Spc42-tdTomato::NATMX, leu2::PURA3::tetR-GFP::LEU2, tetOx224-URA3 (tetOs 11,5 kb to right of CENIV), yil015w::KANMX, his3-11,15::AFB2-HIS3(pEF604), Smc6-AID-9myc-TRP1	This study
SMC5-AID AFB2	YPE4176	Mat a, trp1-1, leu2-3, his3-11, ade2-1, P_MET3_-CDC20::URA3, Spc42-tdTomato::NATMX, leu2::PURA3::tetR-GFP::LEU2, tetOx224-URA3 (tetOs 11,5 kb to right of CENIV), yil015w::KANMX, his3-11,15::AFB2-HIS3(pEF604), Smc1-AID-3myc-TRP1	This study
Rad52 GFP	YEF651	Mata, ura3Δ0, leu2Δ0, his3Δ1, met15Δ0, YML032C-GFP-HIS3MX6	–
Rad52-GFP 2 KE	YEF1647	Mata, ura3Δ0, leu2Δ0, his3Δ1, met15Δ0, YML032C-GFP-HIS3MX6, YOL034W::K624E, YOL034W::K631E	This study
RAD53-HA	YEF954	Mat alpha, ura3-Δ851, trp1-Δ63, leu2Δ1, his3Δ200, ade2-661, yml032c::KANMX, rad53-HA	–
RAD53-HA 2 KE	YEF1882	Mat alpha, ura3-Δ851, trp1-Δ63, leu2Δ1, his3Δ200, ade2-661, yml032c::KANMX, rad53-HA, YOL034W::K624E, YOL034W::K631E	This study
R1	YEF1065	Mat a, ura3Δ0, leu2Δ0/, his3Δ1, lys2Δ202, ade2-661, can1::HPHMX, iyGL117::tetR-mRFP- NATMX, YDR003W::tetO-TEF-URA, HIS3::HIS3-LacI-GFP, YDR095C::256lacO-TEF-LEU2	Herbert et al. 2017
R1 2 KE	YEF1887	Mat a, ura3Δ0, leu2Δ0/, his3Δ1, lys2Δ202, ade2-661, can1::HPHMX, iyGL117::tetR-mRFP- NATMX, YDR003W::tetO-TEF-URA, his3::HIS3-LacI-GFP, yDR095c::256lacO- LEU2, YOL034W::K624E, YOL034W::K631E	This study
R3	YEF1068	Mat alpha, ura3Δ0, leu2Δ0, his3Δ1, lys2Δ202, ade2-661, iyGL117::tetR-mRFP-NATMX, YDR354W::tetO-TEF-URA3 his3::HIS3-LacI-GFP YDR259C::lacO- LEU2	Herbert et al.^38^
R3 2 KE	YEF1706	Mat alpha, ura3Δ0, leu2Δ0, his3Δ1, lys2Δ202, ade2-661, iyGL117::tetR-mRFP NATMX YDR354W::tetO-TEF-URA3 his3::HIS3-LacI-GFP YDR259C::lacO- LEU2, YOL034W::K624E, YOL034W::K631E	This study
Tub2-GFP	YPE257	Mata, ura3-52, trp1Δ63, leu2Δ1, lys2-801, ade2-661, ura3-52::TUB2-GFP-URA3	–
Tub2-GFP SMC5-2 KE	YEF1864	Mata, ura3-52, trp1Δ63, leu2Δ1, lys2-801, ade2-661, ura3-52::TUB2-GFP-URA3, YOL034W::K624E, YOL034W::K631E	This study
Smc5-ndc80-1	YEF1928	Mat alpha, ura3Δ0, trp1Δ63, leu2Δ0, his3Δ1, lys2Δ202, can1::HPHMX,ade2-661, lys2::TetR-GFP-LYS2, yel053c::KanMX:: tetO-URA3, Chr IV 453880 + 453959::KanMX:: mCherry∗I-*Sce*I(cs), ndc80-1	This study
ndc80-1 Smc5-2KE	YEF1930	Mat alpha, ura3Δ0, trp1Δ63, leu2Δ0, his3Δ1, lys2Δ202, can1::HPHMX,ade2-661/,lys2::TetR-GFP-LYS2, yel053c::KanMX:: tetO-URA3, Chr IV 453880 + 453959::KanMX:: mCherry∗I-*Sce*I(cs), YOL034W::K624E, YOL034W::K631E, ndc80-1	This study
L WT sDSB	YEF1203	Mat alpha, ura3Δ0, trp1Δ63, leu2Δ0, his3Δ1, lys2Δ202, ade2-661, lys2::TetR-GFP-LYS2, can1::HPHMX, YEL053C:: KANMX:: tetO-URA3 Chr IV 853996 + 854075:: KanMX:: mCherry∗I-*Sce*I(cs)	García-Fernandez et al.^48^
L 2 KE sDSB	YPE4131	Mat alpha, ura3Δ0, trp1Δ63, leu2Δ0, his3Δ1, lys2Δ202, ade2-661, lys2::TetR-GFP-LYS2, can1::HPHMX, YEL053C::KanMX::tetO-URA3, Chr IV 853996 + 854075:: KanMX:: mCherry∗-I-*Sce*I(cs), YOL034W::K624E, YOL034W::K631E,	This study
C + DSB Local mobility	YPE4133	Mata, ura3Δ0, leu2Δ0, his3Δ1, met15Δ0, YOL034W-13xMYC-URA3, Chr IV 453880 + 453959::KANMX::mCherry∗-I-*Sce*I(cs), ymr008C::kanMX::lacO-LEU2, LacI-HIS3, his3-STOP, pGALISceI-HIS3	This study
C 2 KE - DSB Local mobility	YPE4136	Mata, ura3Δ0, leu2Δ0, his3Δ1, met15Δ0, YOL034W::K624E, YOL034W::K631E, YOL034W-13xMYC-URA3, Chr IV 453880 + 453959::KanMX: mCherry∗I-*Sce*I(cs), ymr008C::kanMX::lacO-LEU2, LacI-HIS3, his3-STOP, p2u-HIS3	This study
C 2 KE + DSB Local mobility	YPE4137	Mata, ura3Δ0, leu2Δ0, his3Δ1, met15Δ0, YOL034W::K624E, YOL034W::K631E, YOL034W-13xMYC-URA3, Chr IV 453880 + 453959::KanMX::mCherry∗-I-*Sce*I(cs), ymr008C::kanMX::lacO-LEU2, LacI-HIS3, his3-STOP, pGALISceI-HIS3	This study
L - DSB DSB Local mobility	YPE4134	Mata, ura3Δ0, leu2Δ0, his3Δ1, met15Δ0, YOL034W-13xMYC-URA3, Chr IV 853996 + 854075::KanMX::mCherry∗-I-*Sce*I(cs), ydr193w::kanMX::lacO-LEU2, LacI-HIS3, his3-STOP, p2u-HIS3	This study
L + DSB Local mobility	YPE4135	Mata, ura3Δ0, leu2Δ0, his3Δ1, met15Δ0, YOL034W-13xMYC-URA3, Chr IV 853996 + 854075::KanMX::mCherry∗-I-*Sce*I(cs), ydr193w::kanMX::lacO-LEU2, LacI-HIS3, his3-STOP, pGALISceI-HIS3	This study
L 2 KE - DSB Local mobility	YPE4138	Mata, ura3Δ0, leu2Δ0, his3Δ1, met15Δ0, YOL034W::K624E, YOL034W::K631E, YOL034W-13xMYC-URA3, Chr IV 853996 + 854075::KanMX::mCherry∗I-*Sce*I(cs), ydr193w::kanMX::lacO-LEU2, LacI-HIS3, his3-STOP, p2u-HIS3	This study
C - DSB ChIP-qPCR	YEF1922	Mata, ura3Δ0, leu2Δ0, his3Δ1, met15Δ0, YOL034W-13xMYC-URA3, Chr IV 853996 + 854075:: kanMX::mCherry∗ISceI(cs), p2u-HIS3	This study
C + DSB ChIP-qPCR	YEF1923	Mata, ura3Δ0, leu2Δ0, his3Δ1, met15Δ0, YOL034W-13xMYC-URA3, Chr IV 853996 + 854075::kanMX:::mCherry∗I-*Sce*I(cs), pGALISceI-HIS3	This study
C 2 KE - DSB ChIP-qPCR	YEF1934	Mata, ura3Δ0, leu2Δ0, his3Δ1, met15Δ0, YOL034W::K624E, YOL034W::K631E, YOL034W-13xMYC-URA3, Chr IV 453880 + 453959:: kanMX:: mCherry∗I-*Sce*I(cs), p2u-HIS3	This study
C 2 KE + DSB ChIP-qPCR	YEF1925	Mata, ura3Δ0, leu2Δ0, his3Δ1, met15Δ0, YOL034W::K624E, YOL034W::K631E, YOL034W-13xMYC-URA3, Chr IV 453880 + 453959:: kanMX:: mCherry∗I*-Sce*I(cs), P_GAL1-10_-*I-SceI*-HIS3	This study
L - DSB ChIP-qPCR	YEF1898	Mata, ura3Δ0, leu2Δ0, his3Δ1, met15Δ0, YOL034W-13xMYC-URA3, Chr IV 853996 + 854075:: kanMX:: mCherry∗I-*Sce*I(cs), p2u-HIS3	This study
L + DSB ChIP-qPCR	YEF1900	Mata, ura3Δ0, leu2Δ0, his3Δ1, met15Δ0, YOL034W-13xMYC-URA3, Chr IV 853996 + 854075::kanMX::mCherry∗I-*Sce*I(cs), P_GAL1-10_-*I-SceI*-HIS3	This study
L 2 KE - DSB ChIP-qPCR	YEF1926	Mata, ura3Δ0, leu2Δ0, his3Δ1, met15Δ0, YOL034W::K624E, YOL034W::K631E, YOL034W-13xMYC-URA3, Chr IV 853996 + 854075::kanMX::mCherry∗I-*Sce*I(cs), p2u-HIS3	This study
L 2 KE + DSB ChIP-qPCR	YEF1920	Mata, ura3Δ0, leu2Δ0, his3Δ1, met15Δ0, YOL034W::K624E, YOL034W::K631E, YOL034W-13xMYC-URA3, Chr IV 853996 + 854075::kanMX::mCherry∗*I-SceI*(cs), P_GAL1-10_-*I-SceI*-HIS3	This study
CEN-SPB	YEF1019	Mat a, his3Δ1 or his3Δ200, lys2Δ202 or 0, cenIV-tetO-URA3, lys2Δ202::tetR-GFP-LYS2, SPC42-mCherry-HIS3	García-Fernandez et al.^35^
CEN-SPB Smc5-2KE	YEF1691	Mat a, his3Δ1 or his3Δ200, lys2Δ202 or 0, cenIV-tetO-URA3, lys2Δ202::tetR-GFP-LYS2, SPC42-mCherry-HIS3, YOL034W::K624E, YOL034W::K631E	This study
Mtw1-3xGFP	YEF1017	Mat a, ura3-1, trp1-1, leu2Δ3,112, his3-11, can1-100, ade2-1, ura3::TUB1-CFP::URA3, YAL034W-A-3xGFP::HIS3, yil015w::KANMX	García-Fernandez et al.^35^
Mtw1-3xGFP Smc5-2KE	YEF4025	Mat a, ura3-1, trp1-1, leu2Δ3,112, his3-11, can1-100, ade2-1, ura3::TUB1-CFP::URA3, YAL034W-A-3xGFP::HIS3, YOL034W::K624E, YOL034W::K631E, yil015w::KANMX	This study
SMC5-3xSTII, CBP-9xHIS-SMC6	D10079	Mata, W303, ura3-1, trp1-1, leu2-3,112, his3-11,15, can1-100, ade2-1, [PSI+], rad5-535, SMC5-3xSTII::TRP1, CBP-9xHIS-SMC6	This study

#### Strain construction

Mutations K624E and K631E in SMC5 were introduced using CRISPR/Cas9 and an 80-bp donor carrying the desired substitutions, as previously described.^3^ Duplexed 20-nucleotide oligonucleotides adjacent to the PAM sequence (AGG) near K631 (oACS13/oACS14) were cloned into a replicative Cas9-KANMX plasmid (pEF562, Addgene plasmid #100955) to generate pEF590. Correct cloning was verified by PCR and sequencing. Equimolar amounts of pEF590 and the 80-bp donor oligonucleotide (oACS69) were used to transform the desired strains, and the resulting *smc5*-*2KE* transformants were verified by sequencing. The same strategy was used to generate *smc5-2KR*, using donor oligonucleotides carrying the appropriate substitutions (oACS270).

Strains used to monitor local DSB mobility were generated as previously described.^3^ Briefly, the I-*SceI* cutting site was introduced at the desired genomic location by replacing a KANMX cassette integrated in intergenic regions of chromosome IV at positions 453880–453959 (strain C) or 853996–854075 (strain L) with an mCherry sequence containing the I-*Sce*I recognition site. LacO repeats located 10 kb from the cutting site were introduced by replacing a KANMX cassette integrated at positions 462252–462602 (strain C) or 844554–844952 (strain L) with BglII-digested pEF222 plasmid (New England Biolabs, R0144). LacI-GFP was introduced using NheI-digested pEF569 (New England Biolabs, R3131). For inducible I-*Sce*I expression, the ARS-CEN-HIS3 plasmid pB07 (obtained from B. Pardo), carrying I-*Sce*I under the control of the GAL1-10 promoter, was used after introduction of a stop codon into the genomic HIS3 sequence using CRISPR/Cas9. Duplexed oligonucleotides oACS204/oACS205 adjacent to the PAM sequence (TGG) in HIS3 and the 80-bp donor oligonucleotide oACS208 were used for this purpose.

Conditional depletion of SMC5 was achieved using an auxin-inducible degron system as previously described.^7^ An AID-9MYC tag was introduced at the C-terminus of SMC5 by homologous recombination using pEF565 and oligonucleotides oACS40/oACS53.^8^ AFB2 was integrated at the HIS3 locus using NheI-digested pEF604 (gift from T. Teixeira), carrying AFB2-HIS3.

#### Media and growth conditions

Cells were grown in SC medium (2 g/L amino acid mix, 20 g/L sugar, and 6.7 g/L yeast nitrogen base) to mid-log phase unless otherwise indicated. Multiple DNA lesions were induced by treatment with 250 μg/mL Zeocin (Thermo Fisher, R25001) for 4 h.^9^ Microtubules were depolymerized using nocodazole dissolved in DMSO for 30 min at the indicated concentration, and depolymerization was verified in parallel by loss of Tub1-GFP signal in strain YPT257.^2^ A single DSB was induced by addition of 2% galactose for 6 h.

For G1 arrest, *bar1Δ* strains were treated with 50 ng/mL alpha-factor (Zymo Research, Y1001M) for 3 h prior to imaging. Metaphase arrest was induced by depletion of CDC20 in PMET3-CDC20 strains using 2 mM methionine for 2 h before imaging.^10^ Prometaphase arrest was induced with nocodazole, using 20 ng/μL for 1 h followed by 10 ng/μL for an additional hour. Auxin-induced degradation of Smc5, Smc6, or Smc1 was performed by adding 1 mM auxin (Sigma, I3750-5G-A), dissolved in 100% ethanol, 1 h before imaging.

### Method details

#### Purification of endogenous Smc5/6 complex and mass spectrometry sample preparation

Purification of the endogenous yeast Smc5/6 complex was performed as described in^11^ with minor modifications. A yeast culture (30L) was grown at 30 °C until it reached an OD_600_ of 0.7–1.0 and then further incubated at 18 °C for 16 h. Cells were collected and washed with H_2_O before being snap frozen. Cells were subsequently thawed on ice in NBE buffer (50 mM potassium phosphate buffer pH 8, 50 mM Tris-HCl pH 8, 500 mM NaCl, 10% glycerol, 0.5% Triton X-100, supplemented with 20 mM imidazole, 2 mM ß Mercapto-Ethanol, 2× protease inhibitors, pH 8). Snap frozen droplets of cell suspensions were lysed using a freezer mill. The resulting powder was then resuspended in NBE buffer and cleared by high-speed centrifugation. The cleared lysate was subjected to two consecutive rounds of Ni-NTA purification. The Ni-NTA columns were washed with 10 CV of NW buffer (50 mM potassium phosphate buffer pH 8, 50 mM Tris-HCl pH 8, 500 mM NaCl, 10% glycerol, 0.5% Triton X-100, supplemented with 60 mM imidazole, 2 mM ß Mercapto-Ethanol, and protease inhibitors, pH 8) and bond proteins were eluted with 5 CV of buffer NE (50 mM Tris HCl pH 8, 500 mM NaCl, 10% glycerol, 0.5% Tween 20, supplemented with 500 mM imidazole, 2 mM bME, and 1× protease inhibitors, pH 8). Eluted fractions were loaded on an SDS-polyacrylamide gel (4–12% Bis-Tris) in 1 × MOPS buffer and ran for 90 min at 150 V before being stained with Coomassie Blue. Peak fractions from both columns were combined and loaded on a 5 mL Strep-Tactin XT column using an AKTA FPLC purification system. The column was washed with 10 CV of buffer SW (50 mM Tris HCl pH 8, 500 mM NaCl, 10% glycerol, 0.5% Tween 20, 2 mM ß Mercapto-Ethanol, 1× protease inhibitors, supplemented with 0.5% Triton X-100, pH 8) and eluted with 12 CV of STE buffer (150 mM NaCl, 10% glycerol in PBS 1×, supplemented with 50 mM Biotin, 2 mM ß Mercapto-Ethanol, 1× protease inhibitors, pH 8). Peak elutions were snap frozen and stored at −80 °C.

#### Microtubule-binding and cell proliferation assays

The affinity of the Smc5 hinge domain (residues 215–885) for microtubule polymers was assessed by pelleting assays as previously described.^12^ Cell proliferation phenotypes of strains expressing wild-type or mutant smc5 alleles were assessed by spotting 5-fold serial dilutions of yeast cultures onto solid medium and scoring growth after incubation for 2–4 days at the indicated temperatures, as previously described.^12^

#### Co-immunoprecipitation

The co-IP was performed as described in (Roy et al., 2024; under revision at eLife) with minor modifications. Yeast cultures (50 mL) were grown at 30°C in YEPD medium to an optical density (OD_600_) of 0.7–0.8. Cells were harvested and lysed with glass beads in co-IP lysis buffer (50 mM HEPES – KOH pH 7.5, 140 mM NaCl, 1% Triton X-100, 0.1% sodium deoxycholate, 1 mM EDTA pH 8.0, 1 mM AEBSF, 10 μM Pepstatin-A, 10 μM E64). The purification resin (StrepTactin XT) was washed with sterile water and PBS-BSA buffer (1× PBS [phosphate buffered saline], 1% BSA) just prior to the co-IP experiment. Cell lysates were cleared by centrifugation (30 min at 13,000rpm) and combined (1 mL) with the washed resin for an overnight incubation (∼12h) at 4°C with gentle rotation (20rpm). The resin was subsequently washed in sequence with 1) co-IP lysis buffer, 2) high salt buffer (50 mM HEPES – KOH pH 7.5, 360 mM NaCl, 1% Triton X-100, 0.1% Sodium deoxycholate, 1 mM EDTA pH 8.0) and 3) TE buffer (10 mM Tris-HCl pH 8.0, 0.1 mM EDTA). The purification resin was finally resuspended in 2× SDS-PAGE sample buffer and heated at 95°C for 5 min to elute purified proteins. The eluted proteins were separated on an SDS-polyacrylamide gel [4–12% Bis-Tris gel ran with 1× MOPS electrophoresis buffer (20 mM MOPS [3-(N-Morpholino) propanesulfonic acid], 5 mM of sodium acetate, 1 mM of disodium EDTA [isodium ethylenediaminetetraacetate dihydrate]) for 2h at 120 V, before being transferred to a membrane and analyzed by immunoblotting using a 1:2,000 dilution of mouse anti-Strep antibody and 1:5,000 dilution of mouse anti-Myc (9E10) antibody (GeneTex 369661).

#### Cell cycle time-course experiments

To assess the kinetics of cell cycle progression, a time course experiment was performed as previously described.^13^ Specifically, yeast strains were grown in YPD medium at 30°C until they reached an OD_600_ of 0.3–0.4. Alpha factor was then added (5 μg/mL) to the medium and, after 1h of growth, a second dose (2 μg/mL) was added to ensure optimal arrest in G1. Once ≥90% of the cells were synchronized in G1, they were released at 30°C in fresh YEPD medium. Alpha factor (5 μg/mL) was added 45 min after the initial release to prevent cell cycle re-entry after completion of the first mitosis. For each time point, 1.5 mL of cells were collected and pelleted for 2 min at 3,000rpm. Cells were then fixed in 1 mL of 70% ethanol overnight at 4°C. Cells were washed twice and resuspended in 0.1M KPO_4_ pH7.0 buffer. DAPI (4′,6- diamidino-2-phenylindole; 2 μg/mL) was used to stain yeast nuclei. Separation of nuclei was manually counted in 100 cells per time-point (*N* = 3). Fluorescent images were taken using a Nikon Eclipse Ti2 inverted microscope, with an 100× oil immersion objective.

#### Flow cytometry

Yeast cells were grown in SC selective medium overnight at 30°C, diluted to O.D_600_ nm = 0.5 in 5 mL next morning for 1h30 at 30°C. If necessary, cells were arrested at G1 or G2/M. 10^7^ cells were centrifuged, fixed in 200 μL 70% ethanol and stored at 4°C for 48h. To perform FACS, cells were centrifuged at 10.000 rpm 5 min at 4°C, washed and resuspended in 1 mL of sodium citrate (50 mM, pH7). 200 μL cells were transferred in polystyrene round-bottom tubes with 20 μL of RNaseA (10 mg/mL Roche, 10109169001) and incubated 1 h at 37°C. Cells were resuspended in 2 mL of sodium citrate (50 mM, pH7) with 2.5 μM of SYTOX™ Green nucleic acid stain (Sigma, S7020). Cells were stained 1h at 4°C in the dark for 1 h. Cells were and analyzed with the Attune NxT Flow cytometer.

#### Microscopy

For microscopy, cells were grown as for FACS. Cells were plated on agarose patches (made of SC medium containing 2% agarose) and sealed using VaLaP (1/3 Vaseline, 1/3 Lanoline and 1/3 Paraffin). Cells were imaged using a widefield microscopy system Nikon Ti-E equipped with the Perfect Focus System (PFS) and a 60× oil immersion objective (Nikon, Plan APO). Andor *Neo* Scmos camera allowed a vision field of 276 × 233 μm with a pixel size of 108 nm. Fluorochromes were excited with 35% LED with a wavelength of 477 nm for GFP or 40% LED with a wavelength of 587 nm for mCherry.

#### Dynamic analysis of chromatin mobility

Analyses of chromatin mobility were performed as described in Herbert et al., 2017. Briefly, GFP tracking was performed by video microscopy for 3 min with an interval time of 100 ms in two dimensions (x,y). A Fiji plugin was used to isolate and extract locus trajectory at each time point throughout the film. After drift correction and signal-to-noise ratio checked with MATLAB scripts, Mean square distances (MSDs) for each trajectory were calculated and concatenated. Power laws to the mean MSD of a population over time intervals between 0.1 and 10s were adapted with MATLAB script and graphs of averaged MSDs were plotted in linear or log/log.

For each single locus trajectory sampled at a time resolution *δt* for an overall time *T*, the MSD at time Δ*t* is calculated as the time-average over the $M=\frac{T-\Delta t}{\delta t}$ frames of the squared displacement performed by the locus between a running initial time *mδt* and the increased time *mδt* + Δ*t*:

$${MSD}_{i}\left(\Delta t\right)=\frac{1}{M}{\sum_{m=1}}^{M}{\left|r_{i}^{⃗}\left(m\delta t+\Delta t\right)-r_{i}^{⃗}\left(m\delta t\right)\right|}^{2}\text{,}$$

For an ensemble of *N* trajectories (cells), one gets an ensemble of *N MSD*_*i*_(Δ*t*) curves; the index *i* therefore refers to *i*-th the trajectory. Each single curve can then be fitted to a *y*(Δ*t*) = *D*Δ*t*^*α*^ function leading to a value of the exponent *α*. The distribution of the ensemble of the values of *α* obtained is shown in Figure 1F. In principle, the sub-diffusion coefficient *D* is also obtained by the same fitting procedure. However, we found that an equivalent, but more robust estimate of the sub-diffusion coefficient is obtained by the trend of the MSD over the very first 15 points, as $D=\frac{{MSD}_{i}\left(15\delta t\right)}{15\delta t}$. The *D* histograms of Figure 1F refer to this definition of the sub-diffusive coefficient.

The MSD can also be averaged over the ensemble of *N* trajectories (cells) as

$$MSD\left(\Delta t\right)=\frac{1}{N}{\sum_{i=1}}^{N}{MSD}_{i}\left(\Delta t\right)=\frac{1}{N}{\sum_{i=1}}^{N}\frac{1}{M}{\sum_{m=1}}^{M}{\left|r_{i}^{⃗}\left(m\delta t+\Delta t\right)-r_{i}^{⃗}\left(m\delta t\right)\right|}^{2}\text{,}$$

corresponding to the curves of Figure 1E.

#### Distance measurements

The analysis of distances between two differently labeled dots (GFP and mCherry) was carried out either for intrachromosomal loci, or for a pericentromeric locus and the SPB. Acquisition of 3D z-stacks consist of 35 frames with z steps of 300 nm, illumination with two consecutive color channels for each plane with an exposure time of 100 ms using a dual band filter set. First, using images from a strain dually labeled for the same locus, chromatic aberration was corrected using the plug-in “Descriptor based registration (2d/3d)”. The 3D z-stacks were then converted to 2D using a maximum intensity projection. The dots were then selected using Fiji and the (x,y) coordinates of the red and green loci calculated using a home-made script on Fiji, taking into account the Gaussian intensity of each fluorescent loci. Comparison of distances between loci was performed using Wilcoxon rank-sum tests.

#### Chromatin immunoprecipitation followed by qPCR (ChIP-qPCR)

Yeast cells were grown either in YPD medium or SC-His Raff 2% overnight at 30°C. Next morning, cells were diluted to a O.D_600_ nm of 0,5 in 45 mL and cells were incubated at 30°C during 1h30 to reach the exponential phase. To check expression of Smc5 in the whole genome, cells were synchronized at G2/M phase using nocodazole (Sigma, M1404). Nocodazole (cci. 2 mg/mL) was added two times during the time of synchronization, 20 ng/μL during a first 1h at 30°C and then 10 ng/μL for an additional hour at 30°C under agitation. Cell-cycle arrest was verified by flow cytometry. In order to check the enrichment of Smc5 around the DSB, DSB was induced to the cell cultures by addition of 2% galactose during 6 h at 30°C. 50 OD were recollected and treated with 1% formaldehyde for 12 min at room temperature with rotation. Crosslinking was quenched by using 2.25 mL of glycine 2.5 M for 5 min with rotation. Then, samples were harvested by centrifugation at 3000 rpm for 5 min and washed twice with cold TBS 1×.

Cells pellet was resuspended in 1.2 mL of lysis buffer (50 mM HEPES-KOH pH7.5, 140 mM NaCl, 1 mM EDTA, 1% Triton X-100, 0.1% sodium deoxycholate) supplemented with 1 mM PMSF, phosphatase inhibitor, anti-protease (Roche, 04693132001) and 300 mg of glass beads (Sigma, G8772) and lysed by MagNA Lyser rotor (Roche, 03359093001). The lysate was obtained by centrifuging an eppendorf tube perforated with a sterile needle at 1000 rpm for 1 min and recovering the extract in a new tube. The extract was transferred to a 1.5 mL falcon tube and sonicated with a PICO sonicator (15×: 30 s ON, 90 s OFF, high intensity) to obtain DNA with a range between 200 and 500 pb. After sonication cells were centrifuged for 10 min at 10.000 rpm at 4°C and 25 μL of the supernatant was saved at −80°C as INPUT. 250 μL of DNA after sonication were incubated with 1 μL of DNA carrier (ST0029, TakaRa Bio), 25 μL of BSA 5% (BP1600, Janssen Pharmacy) and 3,2 μg of anti-MYC antibody (Santa Cruz, Biotechnology, INC) overnight at 4°C. 30 mL of *Dynabeads Pan Mouse IgG* (Thermo Scientific, 11041) were added and incubated at 4°C for 4 h. Beads were washed twice with lysis buffer, twice with lysis buffer +0,36 M NaCl (50 mM HEPES-KOH pH7.5, 360 mM NaCl, 1 mM EDTA, 1% Triton X-100, 0.1% sodium deoxycholate), twice with washing buffer (10 mM Tris-HCl pH8, 0.25 M LiCl, 0.5% NP40, 1 mM EDTA, 0.1% sodium deoxycholate) and once with elution buffer (50 mM Tris-HCl pH8, 10 mM EDTA). The supernatant was removed and the immunoprecipitated chromatin was then eluted by incubation in 250 μL TES buffer (50 Mm Tris-HCl, pH 8.0; 10 mM EDTA; 0,5% SDS) for 15 min at 65°C and the supernatant collected, termed IP. The INPUT was mixed with 120 μL of TES buffer. Both INPUT and IP were de-cross-linked at 65°C with agitation overnight. RNA was degraded by incubation with 2 μL RNase A (10 mg/mL) for 1h at 37°C. Proteins were removed by incubation with 20 μL of proteinase K (10 mg/mL) for 2h at 37°C. DNA was purified by a phenol/Chloroform extraction.

#### Drop test

Overnight cultures were diluted to a O.D_600_ = 0.5 and cells were incubated at 30°C until they reached the exponential phase. Serial 1:5 dilutions were plated starting with a O.D. = 0.2 using the replication plate (R2383-1 EA, Sigma) on YPD with or without 100 nM hydroxyurea (Sigma H8627), 10 μM Camptotehcin (Sigma C9911) and 0.2 μM 4-Nitroquinoline 1-oxide (Sigma N8141). Plates were incubated at 30°C for 72 h.

#### Colony formation assay

To quantify the colonies formed in the absence or presence of an I-*Sce*I induced DSB, cells were grown in -Ade -His + raffinose 2% liquid medium overnight at 30°C. After 24 h, cells were counted in a Malassez chamber and ∼ 100–200 cells were spread on -Ade – His + glucose (control) or + galactose plates (DSB induction). Plates were incubated at 30°C and colonies were counted after 72h.

#### Plasmid maintenance assay

Plasmid stability was assessed using the centromeric plasmid pRS415. WT and smc5-2KE mutant cells carrying the plasmid were grown overnight in YPD 200 cells were spread on YPD and replica plated on SC-Leu plates to determine the fraction of plasmid-retaining cells. Plasmid maintenance efficiency was calculated as the ratio of colony-forming units on SC–Leu versus YPD plates.

#### Western blotting

Electrophoretic protein separation was done using Bolt™ 4–12% Bis-TrisPlus (Thermo Fisher NW04122BOX) in Mini gels tanks (Life Technology) and the electrophoresis buffer was NuPAGE™ MOPS or MES SDS Running buffer (MOPS: Thermo Fisher NP0001; MES: Thermo Fisher, NP0002) for 24–26 min at 220 V. Proteins were transferred in a BioTrace™ nitrocellulose membrane (66485, Pall) in Mini PROTEAN Tetra System tanks (Bio Rad) for 90 min at 100 V. Membranes were blocked with 5% milk in Tween-TBS and then probed with 1/1000 Anti-MYC (Santa Cruz, Biotechnology), 1/1000 anti-HA (Sigma, H3663) or 1/500 anti-yH2A (Abcam, ab15083). Primary antibodies were recognized by secondary antibodies either anti-mouse or anti-rabbit IgG HRP-conjugated diluted 1/10000 (Thermo Fisher A11005 and A31556). Blots were developed using the ECL plus western blotting system (GE Healthcare) during 10–30 s.

#### qPCR-based DSB cutting assay

Cells were grown overnight in SC-Ade-His with 2% Raffinose at 30°C, diluted again in 2% Raffinose until they reached the mid-log phase and then, 2% galactose was added to the culture for I-*Sce*I induction. Cells were collected at 0, 2, 4 and 6 h after galactose induction. DNA was extracted from each sample using phenol/chloroform extraction. For this purpose, cells were centrifuged 1 min at 13000 rpm, the pellet was mixed with 300 mg of glass beads, 200 μL of lysis solution (Triton X-100 2%, SDS 1%, NaCl 100 mM, Tris 10 mM pH 8, EDTA 1 mM) and 200 μL phenol-chloroform-isoamyl alcohol (Sigma, 77617). The mix was vortexed for 10 min at 4°C and centrifuged for 10 min at 13000 rpm at 4°C. 140 μL of the supernatant was transferred into new tubes containing 500 μL ethanol 100% and DNA was precipitated during 1 h at 80°C. The mix was centrifuged for 10 min at 13000 rpm at 4°C. The pellet was washed with 500 μL of ethanol 70% and dried at room temperature resuspended in 40 μL of TE containing 20 μg/μL of RNase (Roche, 10109169001) and incubated 30 min at 42°C. DNA concentrations were quantified using nanodrop.

qPCRs were prepared in a 360-wells plate. All samples were brought to a concentration of 5 ng/μL. In each well, 4 μL of the sample were mixed with 4 μL of Power Track SYBR™ Green master mix (Thermo Fisher A46109) and 0,3 μL of forward and reverses oligonucleotides (final concentration 2.10^-5^ M). Oligonucleotides are listed in Table S1. I-*Sce*I oligonucleotides generated a 187bp fragment that flanks the I-*Sce*I cutting site. ACT1 was used as a control to normalize the samples. The standard curve was performed on actin (control) and I-*Sce*I cutting site (before cut) to evaluate the efficiency of the couple of primers used. Triplicates from each sample were performed and four 1:5 dilutions were performed for the standard curve.

Plate sampling was distributed by the epMotion 5070 robot (Eppendorf), qPCR was performed with QuanStudio5 qPCR machine (Thermo Fisher) under the following conditions: pre-incubation (95°C–5 min – 4.4 °C/min), amplification (45 cycles; 95°C–10 s - 4.4 °C/min; 58°C–10 s - 2.2 °C/min; 72°C–20 s – 4,4 °C/min), melting curve (95°C–5 s - 4.4 °C/min; 60°C–1 min – 2.2 °C/min; 97°C – continuous – 0.06 °C/min; 10 acquisitions/°C) and cooling (40°C–30 s - 2.2 °C/min.). qPCR was analyzed with Thermo Fisher cloud and normalized by the Livak method (2^ΔΔCt^).^14^

#### Polymer modeling

The polymer model was identical to the one used in Liu et al. 2023^15^ which was based on the model of Arbona et al. 2017^1^ but made in Polychrom (https://github.com/open2c/polychrom/tree/master), a python wrapper for polymer simulations using openMM.^5^ The model consisted of a diploid yeast nucleus where each monomer represents 750 bp and is 15 nm in radius. The monomers were connected with harmonic bonds with a spring constant parameterized by a wiggle-distance w such that bond extensions of w lead to bond energies of 1 kT

$$k=\frac{2k_{B}T}{w^{2}}\text{.}$$

From this follows that, in the absence of other forces, the wiggle distance will be equal to $\sqrt{2}$ times the bond standard deviation.

The beads were allowed to interact through the soft “polynomial_repulsive” potential. Stiffness was introduced through a harmonic force on the bond angle which favored alignment. The spring constant was chosen to yield a persistence length of 70 nm which was found optimal by Arbona et al. The nuclear envelope was modeled by a spherical confinement with a 1 μm radius, and all telomeres were attached to the wall using strong harmonic bonds which allowed movement along the periphery but no radial motion. All centromeres were attached to the SPB using harmonic bonds with a 400 nm rest length and a high spring constant, allowing negligible fluctuations in length. The nucleolus was modeled by inserting 112,500 bp at the rDNA region where the size of monomers and bonds were increased by a factor 14. Long simulations were run to collect a set of starting configurations from which the starting conformation for short-time simulations were then randomly chosen upon initialization. The conversion factor between simulation time and experimental time was chosen to match the MSD of the 11 kb loci in the WT condition.

#### AlphaFold3 modeling

To predict potential interactions between the Smc5 hinge domain and candidate binding partners, the Smc5 hinge domain (residues 450–645) was modeled in complex with full-length (Tub1-Tub2) or Shs1 subunit of the septin complex from *Saccharomyces cerevisiae*. Protein sequences were provided in FASTA format, and structural predictions were performed using default AlphaFold3 parameters. The confidence of the predicted interactions was evaluated using the interface predicted TM-score (iPTM), which reflects the likelihood of a specific protein-protein interaction.

### Quantification and statistical analysis

Unless otherwise indicated, data were analyzed using Fiji, MATLAB, GraphPad Prism, and instrument-specific software. Sample size (n), number of independent experiments, and statistical tests are indicated in the corresponding figure legends. Nuclear separation in cell-cycle time-course experiments was scored manually in 100 cells per time point from three independent experiments. For qPCR experiments, reactions were performed in technical triplicate and analyzed using the 2ˆ-ΔΔCt method after validation of primer performance by standard curve analysis.

For chromatin mobility analyses, single-particle trajectories were extracted from live-cell imaging movies and used to calculate mean squared displacement (MSD) curves. MSDs were fitted in MATLAB as described in the Methods to estimate the anomalous exponent (alpha) and the sub-diffusion coefficient (D) from individual trajectories. Unless otherwise indicated, comparisons between MSD-derived distributions or distance measurements were performed using two-sided Wilcoxon rank-sum tests. Other statistical analyses were performed using two-sided Mann–Whitney tests or unpaired two-tailed t-tests, as specified in the corresponding figure legends. Statistical significance is indicated in each figure legend and was defined as ns, not significant; ∗*p* < 0.05; ∗∗*p* < 0.01; ∗∗∗*p* < 0.001; ∗∗∗∗*p* < 0.0001.

Further information on custom analysis pipelines, polymer simulation settings, and imaging workflows is available from the lead contact upon request.

## Supplemental information

Document S1. Figures S1–S11, and Tables S1, S2 and references
