# Supplementary material for: Microtubule-dependent regulation of chromatin dynamics by the Smc5/6 complex
Source: iScience. 2026 Aug 3;29(8):116943. doi: 10.1016/j.isci.2026.116943 (PMC13470446; doi:10.1016/j.isci.2026.116943)
Supplement: Supplementary file 1 [file mmc1.pdf]

iScience, Volume *29*

Supplemental information

Microtubule-dependent regulation of chromatin dynamics by the Smc5/6 complex

Ànnia Carré-Simon, Cecilia Martuzzi, Sarah Isler, Renaud Batrin, Henrik Dahl Pinholt, Timothy Földes, Guillaume Laflamme, Juan Reguera, Maria Barbi, Leonid Mirny, Damien D'Amours, and Emmanuelle Fabre

SUPPLEMENTAL INFORMATION

Figure S1. Mass spectrometry analysis of proteins co-purifying with the yeast Smc5/6 complex and Alphafold3 predictions. The Smc5/6 complex was isolated from crude extracts using Ni-NTA and Strep-Tactin affinity chromatography and purified proteins were subjected to mass spectrometry analysis. **(A)** Silver-stained gel showing the 8 subunits of the yeast Smc5/6 complex obtained after consecutive rounds of Ni-NTA and Strep-Tactin affinity chromatography. The expected positions of Smc5/6 subunits are marked next to the gel. **(B)** Amino acid sequence of the beta chain of yeast tubulin (Tub2). High probability peptide sequences identified in the mass spectrometry analysis are highlighted in yellow. **(C)** Tub2 peptides detected by mass spectrometry. **(D)** Table showing AlphaFold3 predictions of interactions between the Smc5 hinge domain (residues 450–645) and candidate proteins. Only the Tub1/Tub2 complex showed a high-confidence interaction (ipTM = 0.72), while Shs1 displayed low ipTM scores, indicating weak or no predicted interaction**. (E)** Alphafold3 prediction model of Smc5 hinge domain in complex with Tub1/Tub2 dimer. On the left the model is represented by ribbons and colored by its pLDDT according to the color key bar. On the right is shown the prediction alignment error (PAE) plot showing the confidence on the interaction. Regions corresponding to Smc5, Tub1 and Tub2 are indicated. **(F)** Microtubule polymer binding assay. Coomassie blue-stained gels showing the binding of the hinge region of Smc5 (residues 215–885) to the microtubule (MT, µM) polymer fraction (pellet; P) after centrifugation. Note how Smc5 hinge domain progressively transferred from the depolymerized tubulin fraction (supernatant; S) to the polymer fraction (P) as the MT concentration increased and eventually bound all available Smc5 protein (top gel). In contrast, the Smc5-2KE mutant (lower gel) showed strongly reduced affinity for MT polymers, as evidenced by the substantial fraction of the hinge domain remaining in the supernatant fraction at the highest concentration of MT shown in the gel (4 µM).

Figure S2. Smc5-2KE mutation does not affect cell growth nor cell cycle. (A) Experimental protocol used to synchronize cells in G1 using alpha factor. **(B)** Percentage of budded and late anaphase cells at each time point, respectively. **(C)** Cell synchronization monitored by flow cytometry. **(D)** Images showing examples of G1 (**left**), metaphase (**middle**) and anaphase (**right**) cell morphologies monitored in yeast cultures expressing Smc5 *or* smc5-2KE. Prior to

imaging, cells were fixed, washed and stained with DAPI to enable nuclei visualization. Scale bars represent 5µm. **(E)** Growth phenotypes on solid medium in the presence of DNA-damaging agents — 100 mM hydroxyurea (HU), 10 µM camptothecin (CPT), or 4-nitroquinoline-1-oxide (4NQO). 1/10 serial dilutions were spotted and photographed after 48h. **(F)** DNA damage sensitivity of smc5-2KE in different strain backgrounds. Ten-fold serial dilutions of WT and smc5-2KEcells in BY-derived and W303-derived backgrounds were spotted onto YPD plates containing 100 mM HU or 4NQO at the indicated concentrations, or no drug as a control, and photographed after 48 h. **(G)** Growth phenotypes on solid medium at different temperatures. 1/10 serial dilutions were spotted and photographed after 48h **(H)** Chromosomal association of Smc5-MYC at selected loci in the indicated strains, as determined by ChIP-qPCR. Cells were arrested in G2/M prior to sample collection. The Y-axis represents the amount of DNA in the ChIP fraction relative to input. Primers were designed to amplify the centromeres of chromosomes 9 (Cen9) and 13 (Cen13), the *rDNA* non-transcribed spacer 1 (Nts1), telomere 15 (Tel15). *ACT1* gene is used as a control. Each dot represents an independent ChIP experiment; bars indicate the mean, and error bars the standard deviation. Statistical significance was assessed using a two-sided Mann–Whitney test; ns, not significant ($p > 0.05$).

Figure S3. The negative charges of K624E and K631E, and not post-translational modifications, lead to increased chromatin mobility along the chromosomal arm in smc5-2KE with no effect on chromatin cohesion. **(A)** Table summarizing the different conditions with values extracted from the MSD: MSD at 10 s, the anomalous exponent (alpha), the sub-diffusive coefficient (D) and the number of cells analyzed. **(B)** MSDs as a function of increasing time intervals on a log-log scale in the WT (blue) and in the *smc5*-2KE mutant (green) for a locus at different positions of chromosome IV (11kb, 90 kb and 730 kb, from left to right). Statistical significance was assessed using a two-sided Wilcoxon rank-sum test; *$p < 0.05$; **$p < 0.0001$. **(C)** Ratio of MSD at 10 s between *smc5-2KE* versus WT at different positions of the chromosome IV. **(D)** MSDs as function of time in the WT, *smc5-2KR* and *smc5-2KE* mutants. Statistical significance was assessed using a two-sided Wilcoxon rank-sum test; ns, not significant; ***$p < 0.0001$. **(E-F)** Cohesion status was assessed based on the number of GFP foci separation of tetO/TetR-GFP marker at ~11 kb from the centromere of chromosome IV in metaphase. Cells were synchronized by Cdc20 depletion. **(E)** WT Smc5 or the *smc5-2KE*

mutated cells, and **(F)** cells in which Smc1 was tagged with an auxin-inducible degron. Cells with a single GFP (yellow) dot were scored as having cohesive sister chromatids, while cells with two GFP foci (purple) were scored as having defective cohesion. For each biological replicate, 200 cells were scored. Data show means of three replicates, with error bars representing ± sd. Statistical significance was assessed using an unpaired two-tailed t-test; ns, not significant; *p < 0.01.

Figure S4. Smc5-2KE mutation increases chromatin mobility throughout the cell cycle. (A) Experimental protocol used to synchronize cells in G1 with 50 ng/ml of alpha factor (**left**). WT and *smc5*-2KE MSDs at 11 and 90 Kb at G1. Wilcoxon rank-sum test results between distributions, with the p-value *** (p<0.0001) (**right**). **(B)** Experimental protocol used to synchronize in G2/M cells carrying the P_{MET3}-*CDC20* with 2 mM of methionine (**left**). WT and *smc5-2KE* MSDs at 11 and 23 Kb at G2/M phase. Statistical significance was assessed using a two-sided Wilcoxon rank-sum test; ***p < 0.0001 (right). (**C**) Table summarizing the different conditions with values extracted from the MSD: MSD at 10 sec, the alpha, the sub-diffusive coefficient (D) and the number of cells analyzed.

Figure S5. The *smc5-2KE* mutant does not induce spontaneous DNA damage. (A) Rad52-foci (protein involved in HR) in WT and *smc5-2KE* mutant (2KE). Three independent experiments were conducted with the number of cells counted as follows: N_{WT}=608, N_{2KE}=1388, $N_{WT\ ZEO}$=650, $N_{2KE\ ZEO}$=509. Statistical significance was assessed using an unpaired two-tailed t-test; ns, not significant; ***p < 0.0001. (**B**) Representative results from immunoblotting showing the phosphorylation (Ph) status of Rad53 in WT, *smc5-2KE* cells and in response to Zeocin treatment (zeo). Actin was used as a loading control. Blots are representative of three experiments. Protein levels are represented as mean ratio values quantified from the phosphorylated form of Rad53 versus actin. Statistical significance was assessed using a two-sided Mann–Whitney test; ns, not significant. **(C)** Representative results from immunoblotting showing the phosphorylation of H2A (γH2A) in WT, *smc5-2KE* cells and in response to Zeocin treatment. Actin was used as a loading control. Blots are representative of three experiments (**left**). Protein levels are represented as mean ratio values quantified from protein γH2A versus actin (**right**). Statistical significance was assessed using a two-sided Wilcoxon rank-sum test; ***p < 0.0001.

Figure S6. The Smc5 or Smc6 degradation decreases chromatin dynamics. (A) Experimental protocol used to synchronize cells in metaphase with 2 mM of methionine cells carrying the P_{MET3}-*CDC20*, followed by degradation of SMCs with 1mM auxin for 1 hour prior to imaging. Auxin being diluted in ethanol (EtOH), controls with EtOH only were performed **(B)** Growth phenotypes on solid medium with or without Auxin in WT, Smc5-AID, Smc6-AID and Smc1-AID **(C)** Representative results from immunoblotting showing the degradation of the Smc5, Smc6 and Smc1 upon 30 min. of treatment with 1 mM auxin. Actin was used as a loading control. The arrows indicate the molecular weights of 150 kDa and 50 kDa at the top and bottom of the gels respectively. Blots are representative of three experiments. **(D-E)** MSDs as a function of increasing time intervals in the absence of auxin (dark blue) and in the presence of Auxin (light blue) in a locus at 11kb from the CEN4 in **(D)** Smc5-AID, Smc6-AID and **(E)** Smc1-AID. Statistical significance was assessed using a two-sided Wilcoxon rank-sum test; ***$p < 0.0001$. **(F)** Table summarizing the different conditions with values extracted from the MSD: MSD at 10 sec, the anomalous exponent (alpha), the sub-diffusive coefficient (D) and the number of cells analyzed.

Figure S7. Weakening the microtubule tether to the kinetochore gradually abolishes position-dependent mobility. Sweep of the wiggle distance (wd) of the bond connecting the centromere to the spindle pole body (See material and methods for a mathematical definition of wd. In the absence of other forces it is $\sqrt{2}$ times the bond standard deviation). **(A)** MSD of loci 11 kb from the centromere. **(B)** MSD of loci 90 kb from the centromere. Candidate loci are selected as in Figure 4 and wild type (WT) refers to the simulation in Figure 4A.

Figure S8. Increasing chromatin stiffness increases mobility globally. Sweep of chromatin stiffness by increasing the stiffness of the bond angle potential by up to a factor two. **(A)** Loci 11kb from the centromere **(B)** Loci 90kb from the centromere. Candidate loci are selected as in Figure 4 and wild type (WT) refers to the simulation in Figure 4A.

Figure S9. Combining chromatin stiffness and loosening the kinetochore tether further increases mobility in the pericentromeric region. Sweep of chromatin stiffness as in Figure S7 but with a wiggle distance (wd) of 105 nm on the bond connecting the centromere and the spindle pole body. Candidate loci are 11 kb from the centromere and selected as in Figure 4. Wild type (WT) refers to the simulation in Figure 4A.

Figure S10. Clustering of centromeres is decreased in the smc5-2KE mutant. Representative fluorescence microscopy images showing. Mtw1-3xGFP in wild-type (WT) and smc5-2KE mutant cells. Cells with or without nocodazole treatment. Scale bar: 5 µm

Figure S11. Smc5-2KE mutation affects HR at pericentromeres without affecting Smc5/6 chromatin loading. **(A)** I-*Sce*I efficiency (%) after 0h, 2h, 4h and 6h after inducing the I-*Sce*I with galactose in Smc5 WT and *smc5-2KE* for strains with a DSB at centromeric (C) or luminal (L) positions. qPCR was done using primers flanking the I-*Sce*I cutting site and actin was used as a loading control. The error bars represent the standard deviation of three independent experiments. It should be noted that the MYC-tagged version of Smc5 contains an I-*Sce*I site, which results in the formation of two double-strand breaks (DSBs) when I-*Sce*I is induced in this strain background without, however, affecting the efficiency of ChIP-qPCR**. (B)** Proportion of cells retaining a centromeric plasmid after growth in nonselective conditions. Statistical significance was assessed using a two-sided Mann–Whitney test; ns, not significant. **(C,D)** Chromosomal association of Smc5-MYC and Smc5-2KE-MYC at selected chromosomal positions as determined by ChIP-qPCR. Chromosomal positions from the DSB are indicated in the X-axis. Unpaired two-tailed t-test was used to analyze differences between groups, with “ns” for not significant ($P>0.05$) and * for $P<0.01$.

TABLES

Table S1: primers used in this study

Table S2: plasmids used in this study

REFERENCES CITED IN SUPPLEMENTAL INFORMATION

81. Adhikary, H., & D’Amours, D. (2025). Production and Purification of the Human SMC5/6 Core Complex from S. cerevisiae (Vol. 2991). Springer US.
82. Nishimura, K. & Kanemaki, M. T. (2014). Rapid Depletion of Budding Yeast Proteins via the Fusion of an Auxin-Inducible Degron (AID). *Curr Protoc Cell Biol* 64, 20.9.1-20.9.16.
83. Morawska, M. & Ulrich, H. (2013). An expanded tool kit for the auxin-inducible degron system in budding yeast. *Yeast* 30, 341–351.

84. Ratsima, H. *et al.* (2011). Independent modulation of the kinase and polo-box activities of Cdc5 protein unravels unique roles in the maintenance of genome stability. *Proc Natl Acad Sci U S A* 108.

85. Livak, K. J. & Schmittgen, T. D. (2001). Analysis of Relative Gene Expression Data Using Real-Time Quantitative PCR and the 2−ΔΔCT Method. *Methods* 25, 402–408.

86. Liu, S. *et al.* (2023). In vivo tracking of functionally tagged Rad51 unveils a robust strategy of homology search. *Nat Struct Mol Biol* 30, 1582–1591.

87. Arbona, J. M., Herbert, S., Fabre, E. & Zimmer, C. (2017). Inferring the physical properties of yeast chromatin through Bayesian analysis of whole nucleus simulations. *Genome Biol* 18.

88. Eastman, P. *et al.* (2017). OpenMM 7: Rapid development of high performance algorithms for molecular dynamics. *PLoS Comput Biol* 13, e1005659.

Figure S1

A

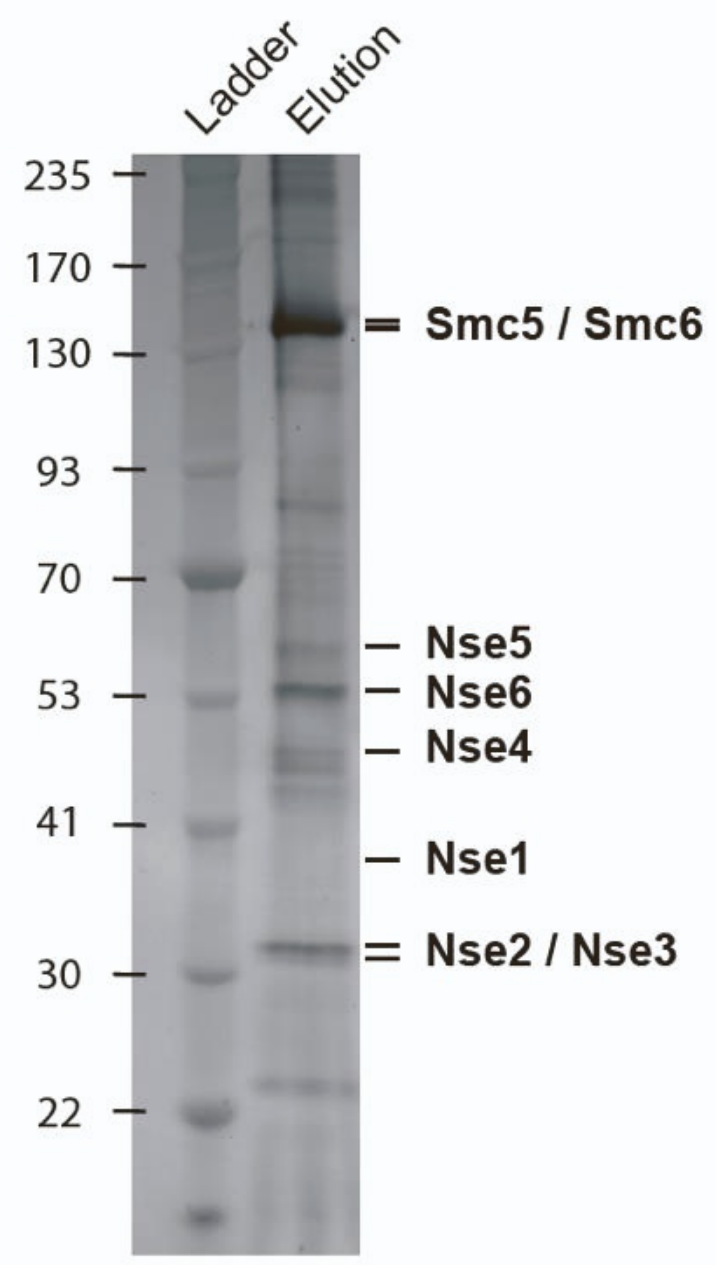

B

Tubulin Beta Chain – *Saccharomyces cerevisiae*

MREIIHISTG QCGNQIGAAF WETICGEHGL DFNGTYHGHD
DIQKERLNVY FNEASSGKWV PR**SINVDLEP** **GTIDAVR**NSA
IGNLFRPDNY IFGQSSAGNV WAKGHYTEGA ELVDSVMDVI
RREAEGCDSL QGFQITHSLG GGTGSGMGTL LISKIREEFP
DRMMATFSVL PSPKTSDTVV EPYNATLSVH QLVEHSDETF
CIDNEALYDI CQRTLKLNQP SYGDLNNLVS SVMSGVTTSL
R**YPGQLNSDL** **R**K**LAVNLVPF** **PR**LHFFMVGY APLTAIGSQS
FRSLTVPELT QQMFDAKNMM AAADPRNGRY LTVAAFFRGK
VSVKEVEDEM HKVQSKNSDY FVEWIPNNVQ TAVCSVAPQG
LDMAATFIAN STSIQELFKR VGDQFSAMFK RKAFLHWYTS
EGMDELEFSE AESNMNDLVS EYQQYQEATV EDDEEVDENG
DFGAPQNQDE PITENFE

C

Peptide sequences	Probability
(R)SINVDLEPGTIDAVR(N)	100%
(R)YPGQLNSDLR(K)	98%
(K)LAVNLVPFPR(L)	98%

D

Smc5 Region	Interacting Protein	Protein Complex	ipTM	pTM	Confidence
450–645	Shs1	Septin complex	0.18	0.48	Low
450–645	Tub1/Tub2	Microtubule (α/β-tubulin)	0.72	0.8	High

E

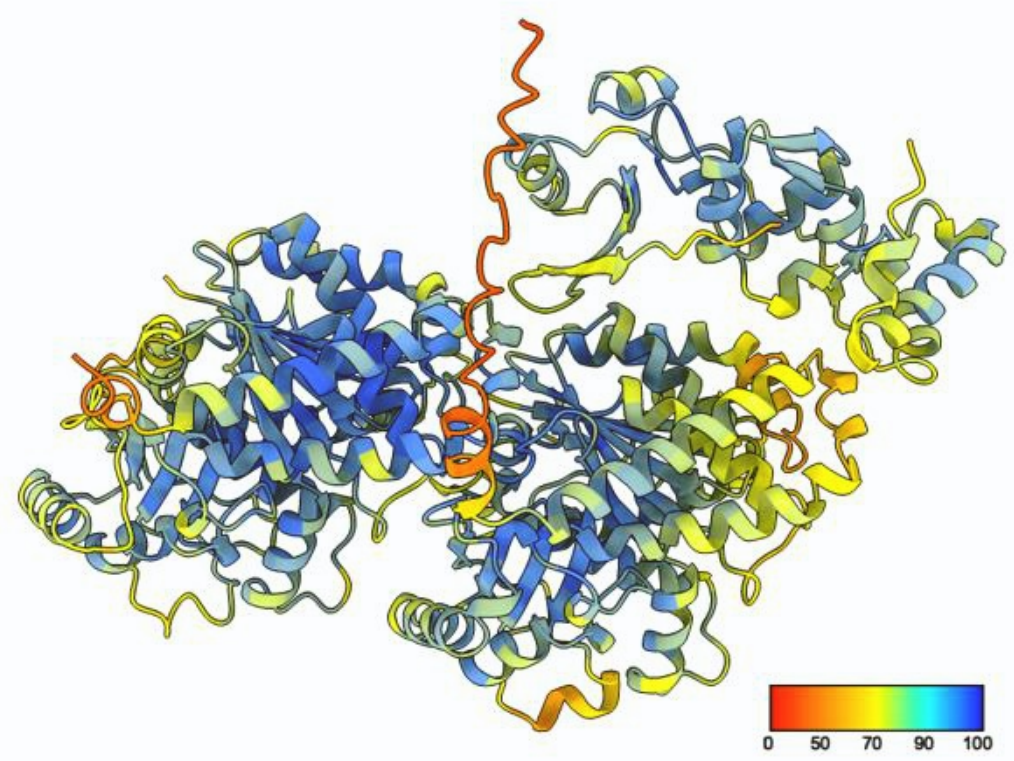

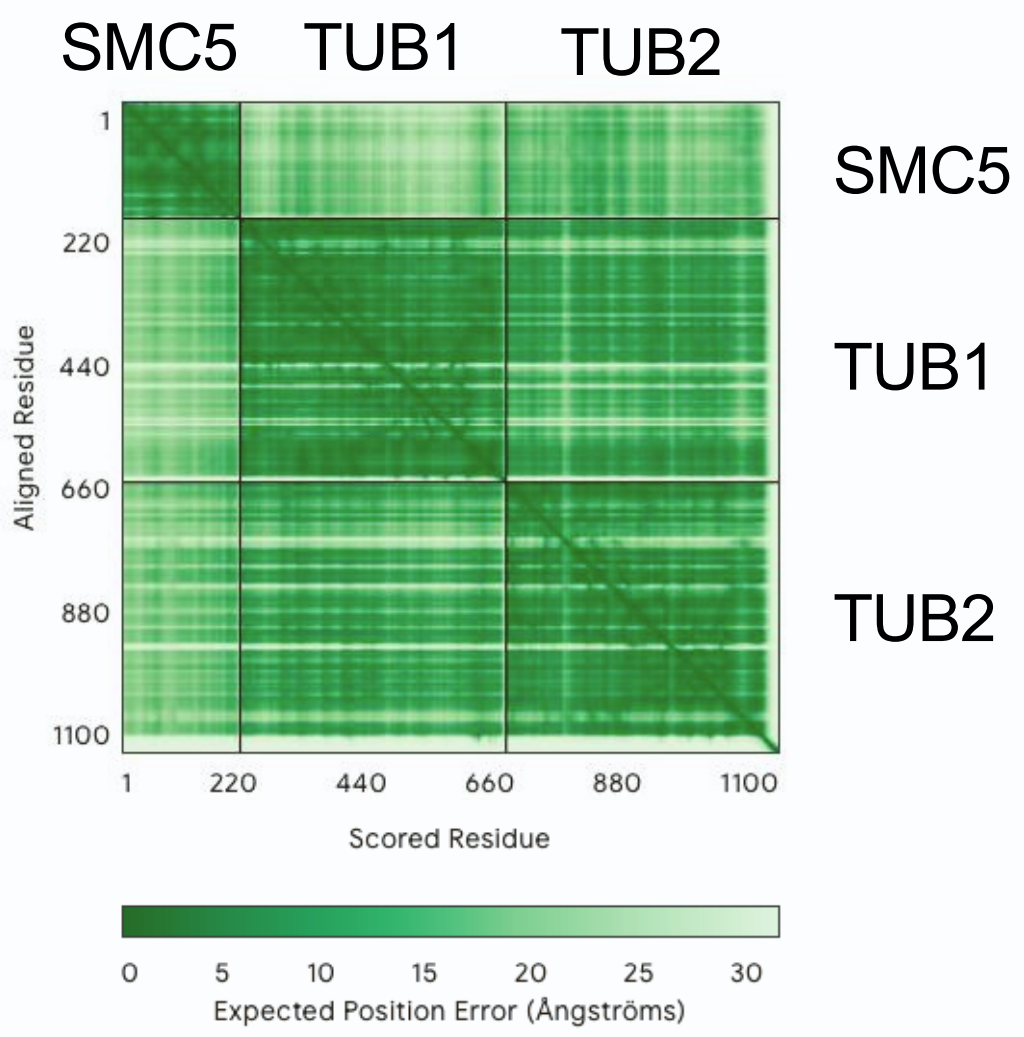

F

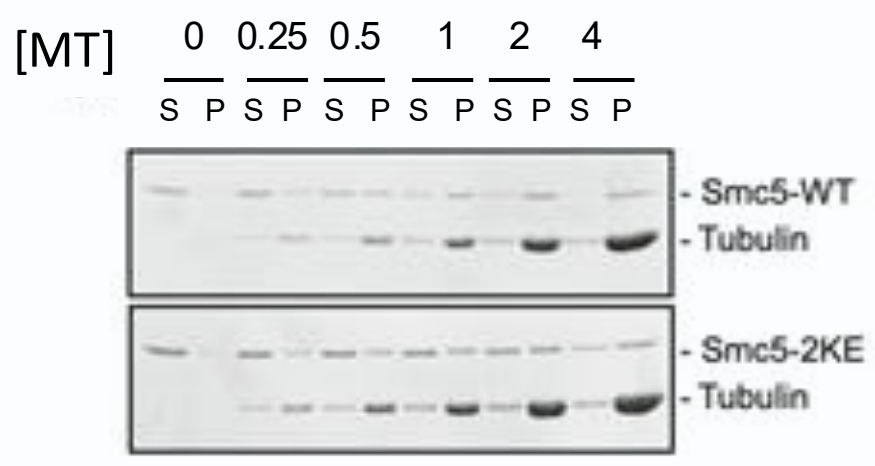

Figure S2

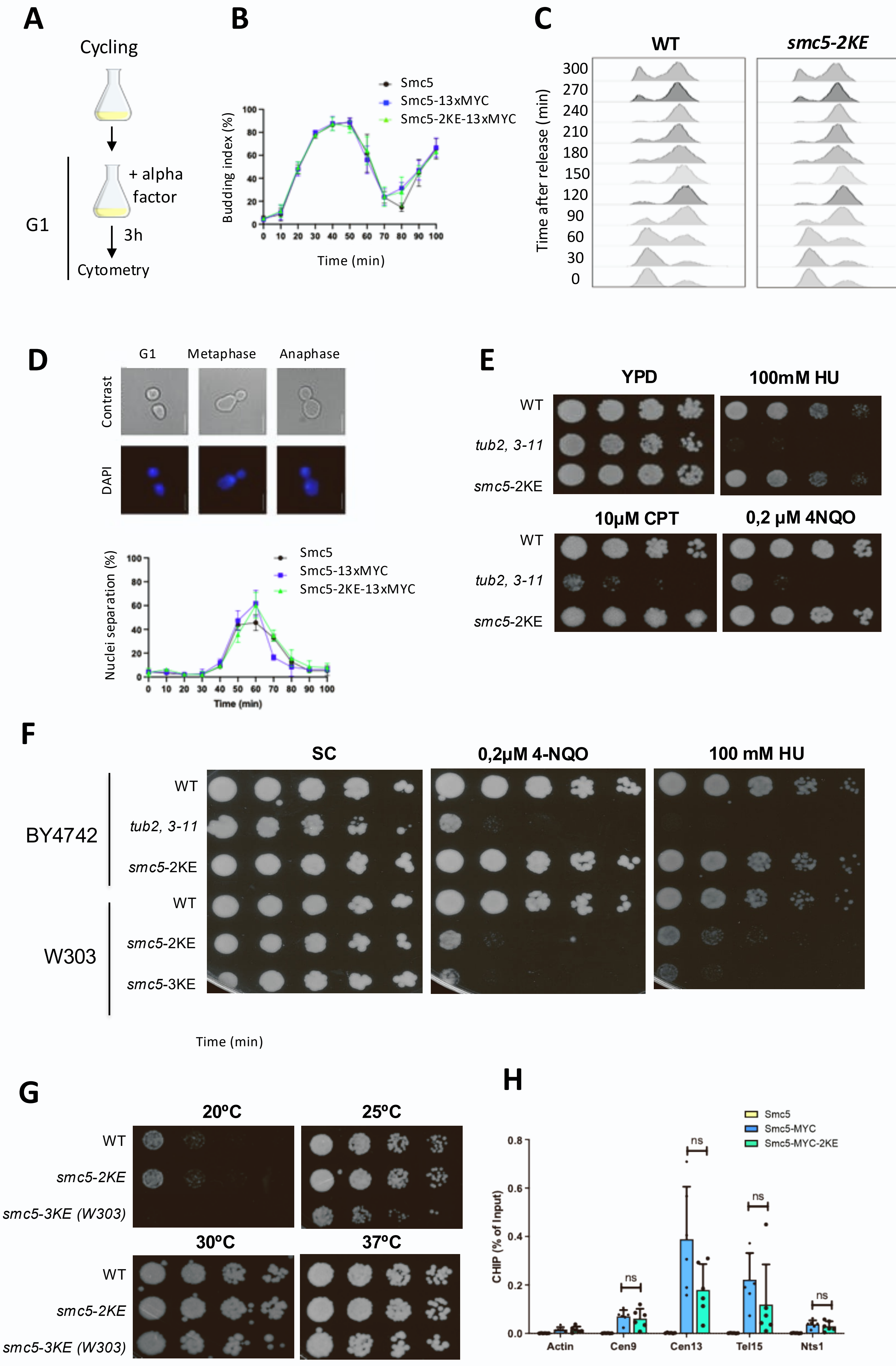
A
Cycling
+ alpha factor
G1
3h
Cytometry
B
Smc5
Smc5-13xMYC
Smc5-2KE-13xMYC
Budding index (%)
Time (min)
C
WT
smc5-2KE
Time after release (min)
300
270
240
210
180
150
120
90
60
30
0
D
G1
Metaphase
Anaphase
Contrast
DAPI
Nuclei separation (%)
Smc5
Smc5-13xMYC
Smc5-2KE-13xMYC
Time (min)
E
YPD
100mM HU
WT
tub2, 3-11
smc5-2KE
10μM CPT
0,2 μM 4NQO
F
SC
0,2μM 4-NQO
100 mM HU
BY4742
W303
WT
tub2, 3-11
smc5-2KE
smc5-3KE
Time (min)
G
20°C
25°C
30°C
37°C
WT
smc5-2KE
smc5-3KE (W303)
H
Smc5
Smc5-MYC
Smc5-MYC-2KE
CHIP (% of Input)
ns
Actin
Cen9
Cen13
Tel15
Nts1

Figure S3

A

Tracked locus	Smc5 allele	MSD at 10 sec (10^{-2})	Alpha	D (10^{-3})	Cell number
11 kb	WT	3.35	0.61	2.0	930
	smc5-2KE	4.57	0.60	2.7	1180
90 kb	WT	6.97	0.59	2.9	2268
	smc5-2KE	8.25	0.58	3.3	3047
730 kb	WT	6.97	0.60	4.8	1042
	smc5-2KE	7.52	0.61	5.2	1566
90 kb	*smc5-2KE*	7,63	0.62	4.4	1557
	smc5-2KR	6.49	0.61	4.0	1290

C

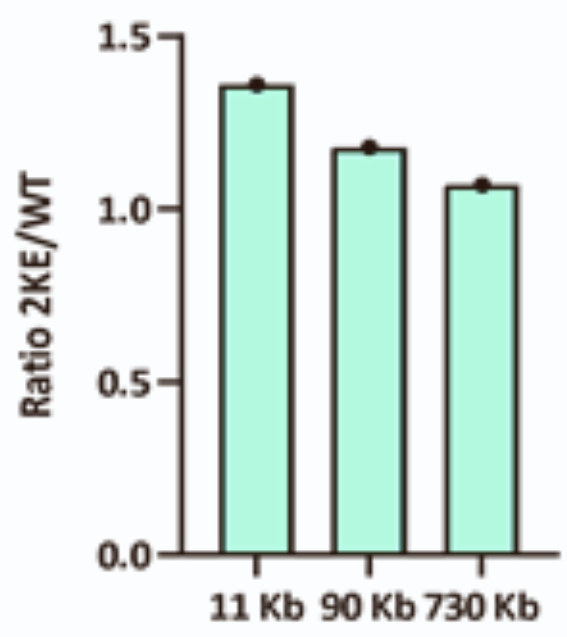

D

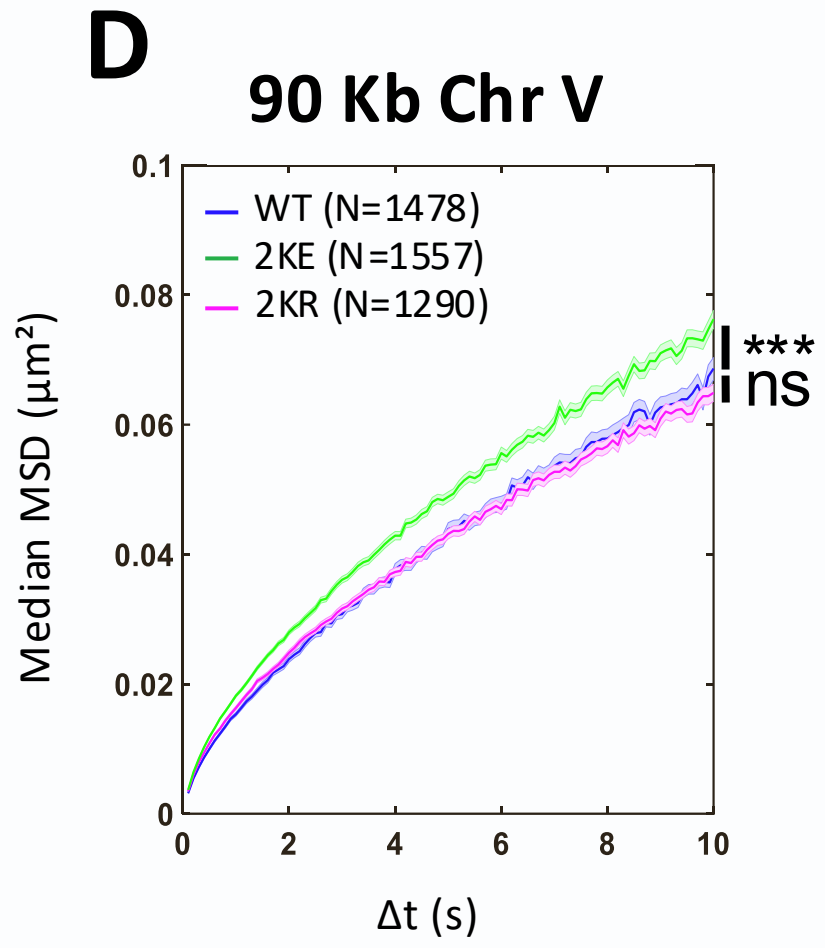

B

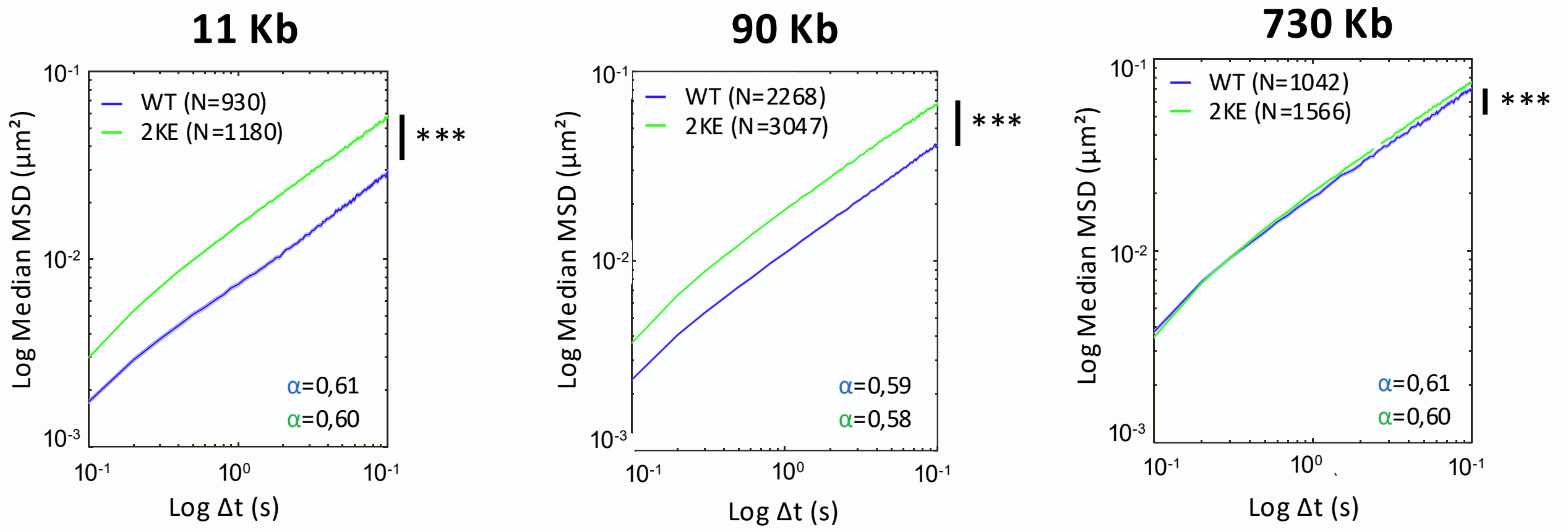

E

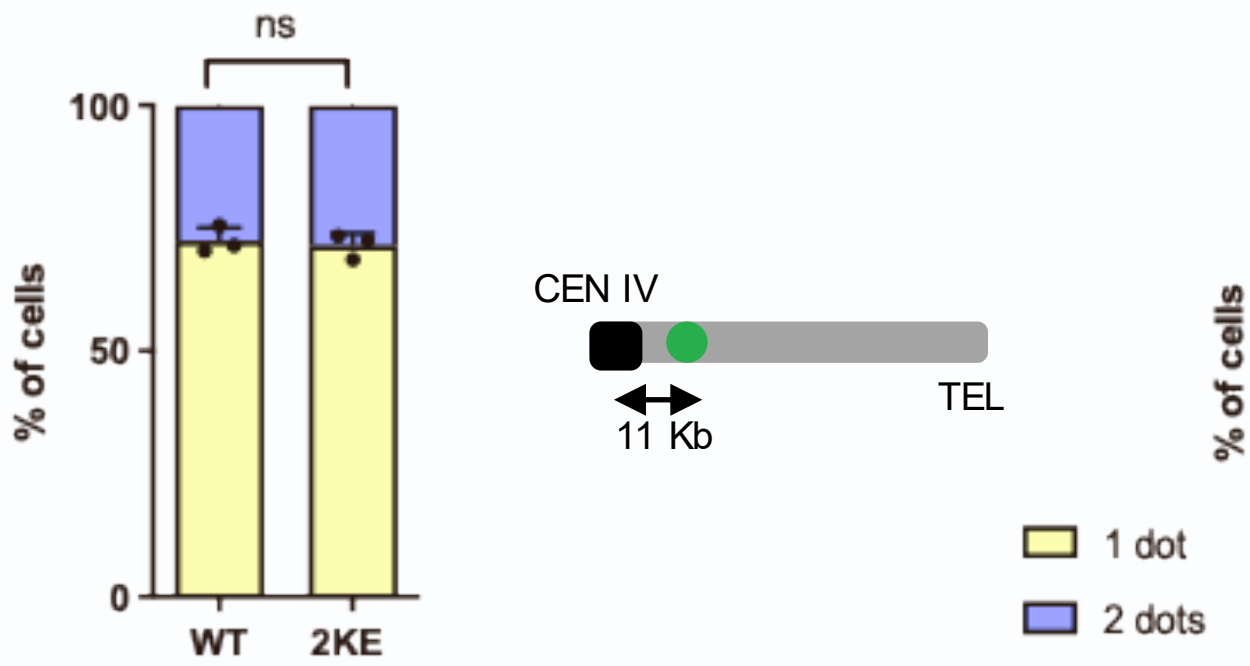

F

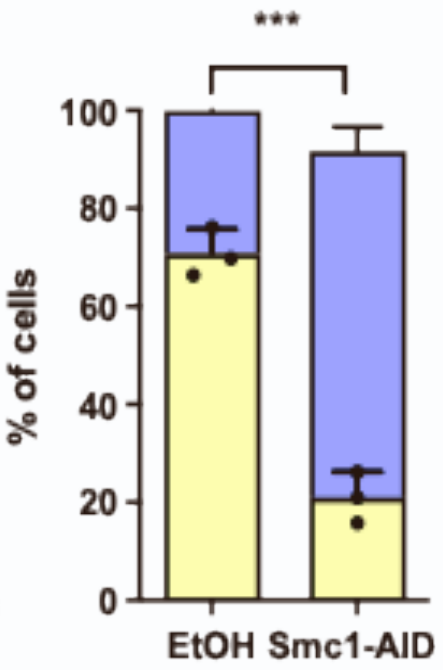

Figure S4

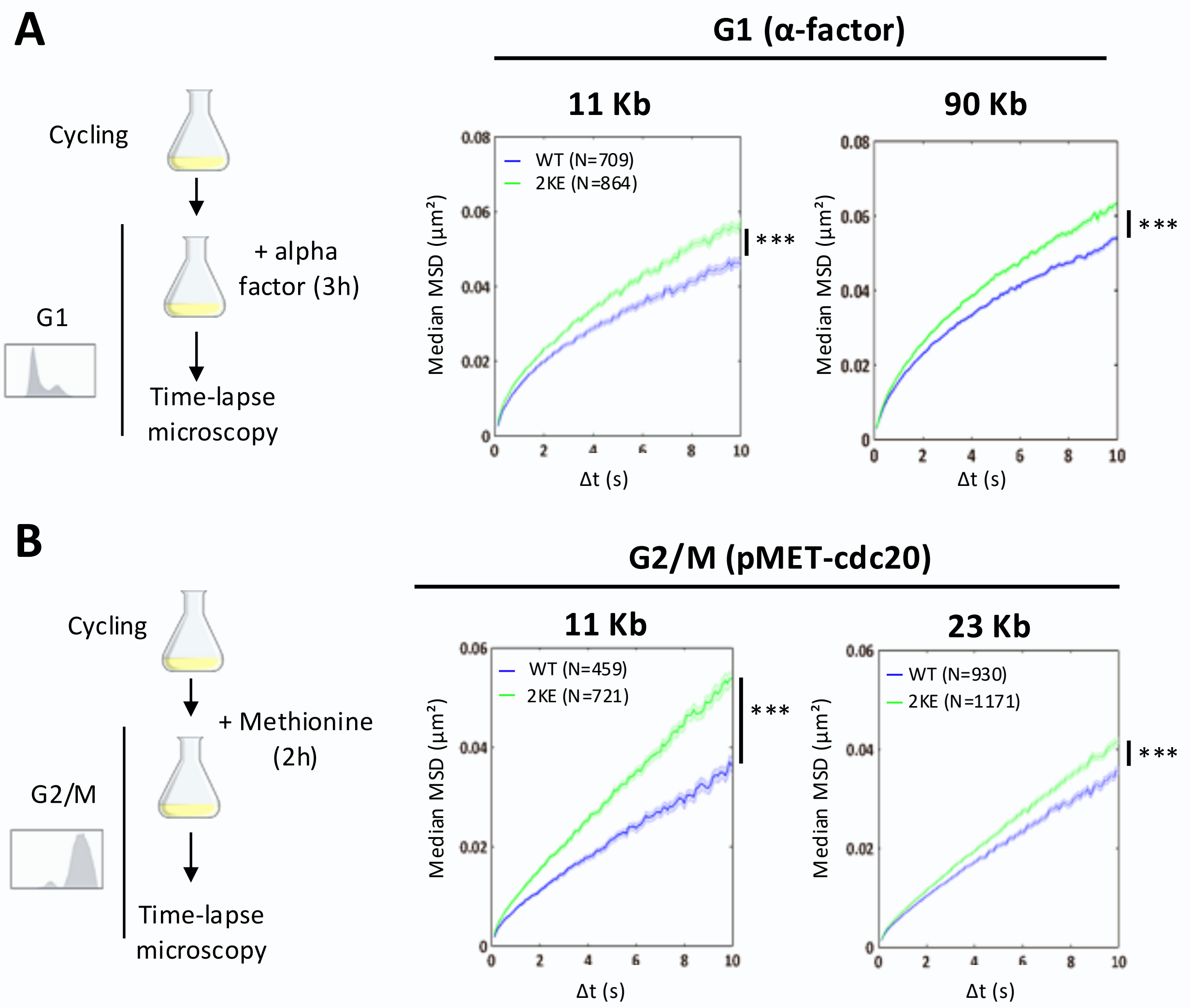

C

Cell phase	Tracked locus	Strain	MSD at 10 sec (10^{-2})	Alpha	D (10^{-3})	Cell number
G1	11 kb	WT	4.56	0.56	3.3	709
		smc5-2KE	5.72	0.56	3.8	864
	90 kb	WT	5.34	0.56	3.2	1838
		smc5-2KE	6.32	0.58	3.5	1623
G2/M	11 kb	WT	3.60	0.64	1.9	459
		smc5-2KE	5.38	0.67	2.5	865
	23 kb	WT	3.58	0.67	1.7	930
		smc5-2KE	4.18	0.69	1.8	1757

Figure S5

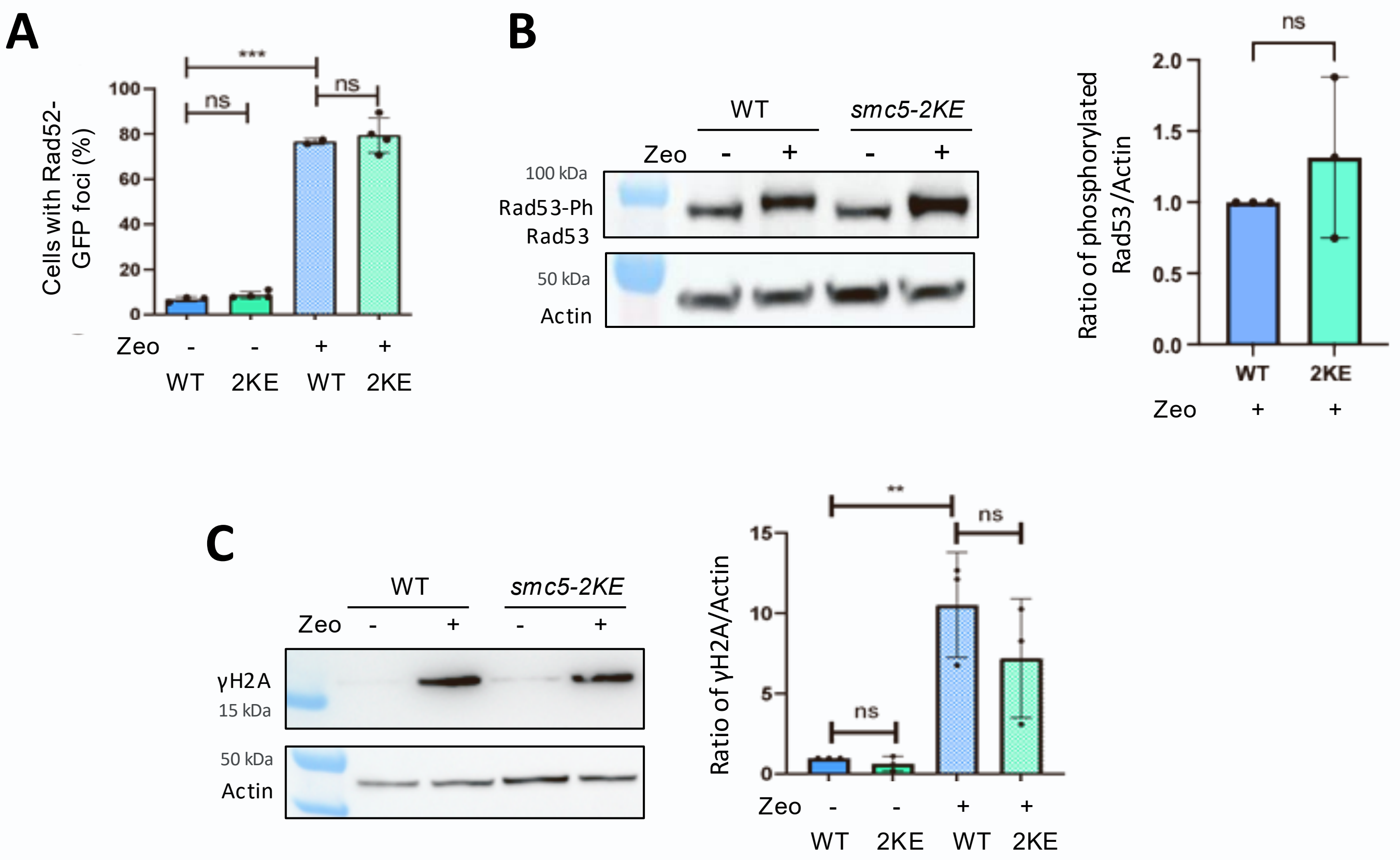

A
Cells with Rad52-GFP foci (%)
***
ns
ns
Zeo - - + +
WT 2KE WT 2KE
B
WT
smc5-2KE
Zeo - + - +
100 kDa
Rad53-Ph
Rad53
50 kDa
Actin
Ratio of phosphorylated Rad53/Actin
ns
WT 2KE
Zeo + +
C
WT
smc5-2KE
Zeo - + - +
γH2A
15 kDa
50 kDa
Actin
Ratio of γH2A/Actin
**
ns
ns
Zeo - - + +
WT 2KE WT 2KE

Figure S6

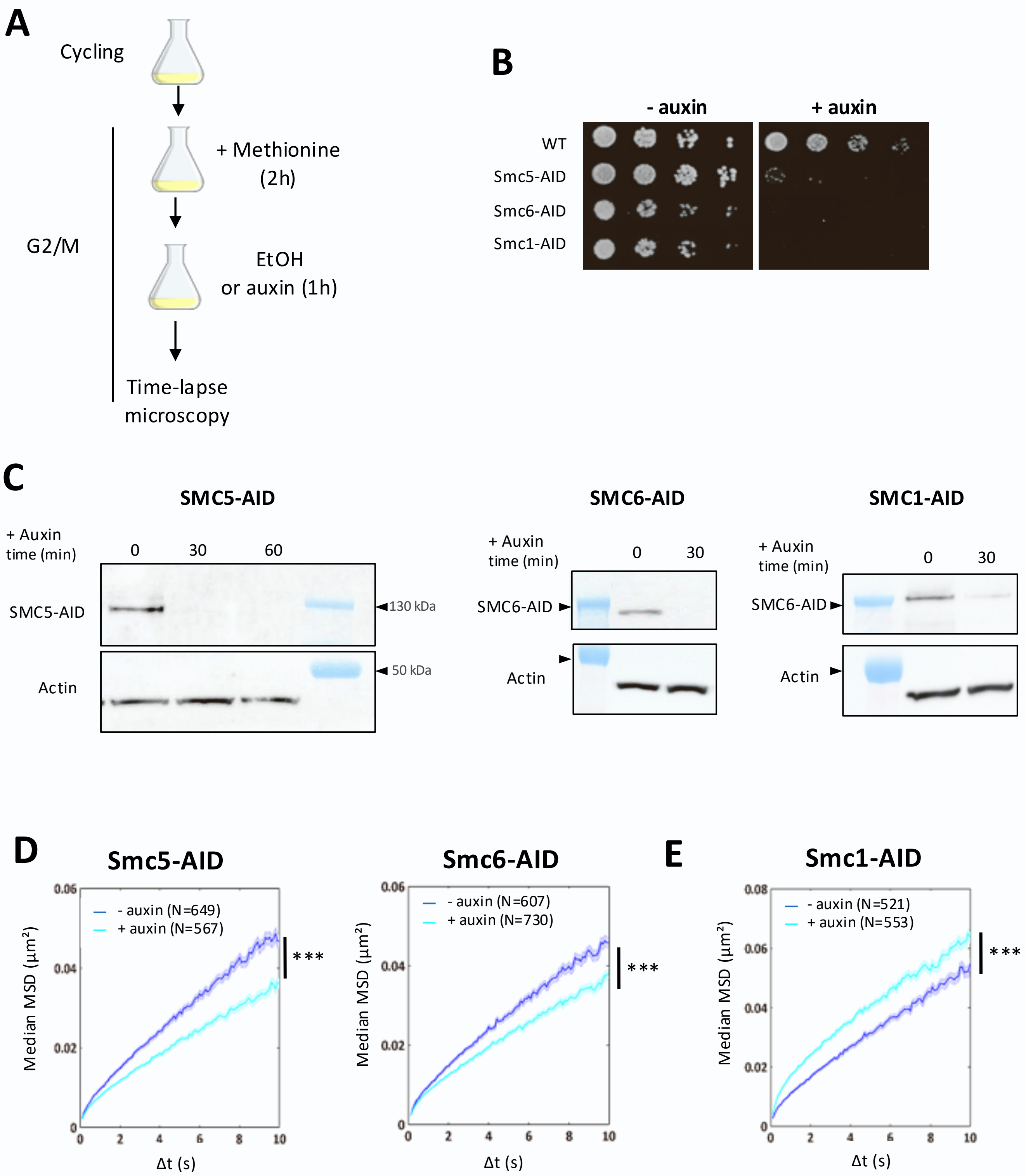

F

	Auxin	MSD at 10 sec (10^{-2})	Alpha	D (10^{-3})	Cell number
**Smc5-AID**	-	4.68	0.63	2.5	649
	+	3.62	0.61	1.9	567
**Smc6-AID**	-	4.59	0.64	2.5	607
	+	3.84	0.62	2.2	730
**Smc1-AID**	-	5.47	0.64	2.7	521
	+	6.34	0.58	4.1	553

Figure S7

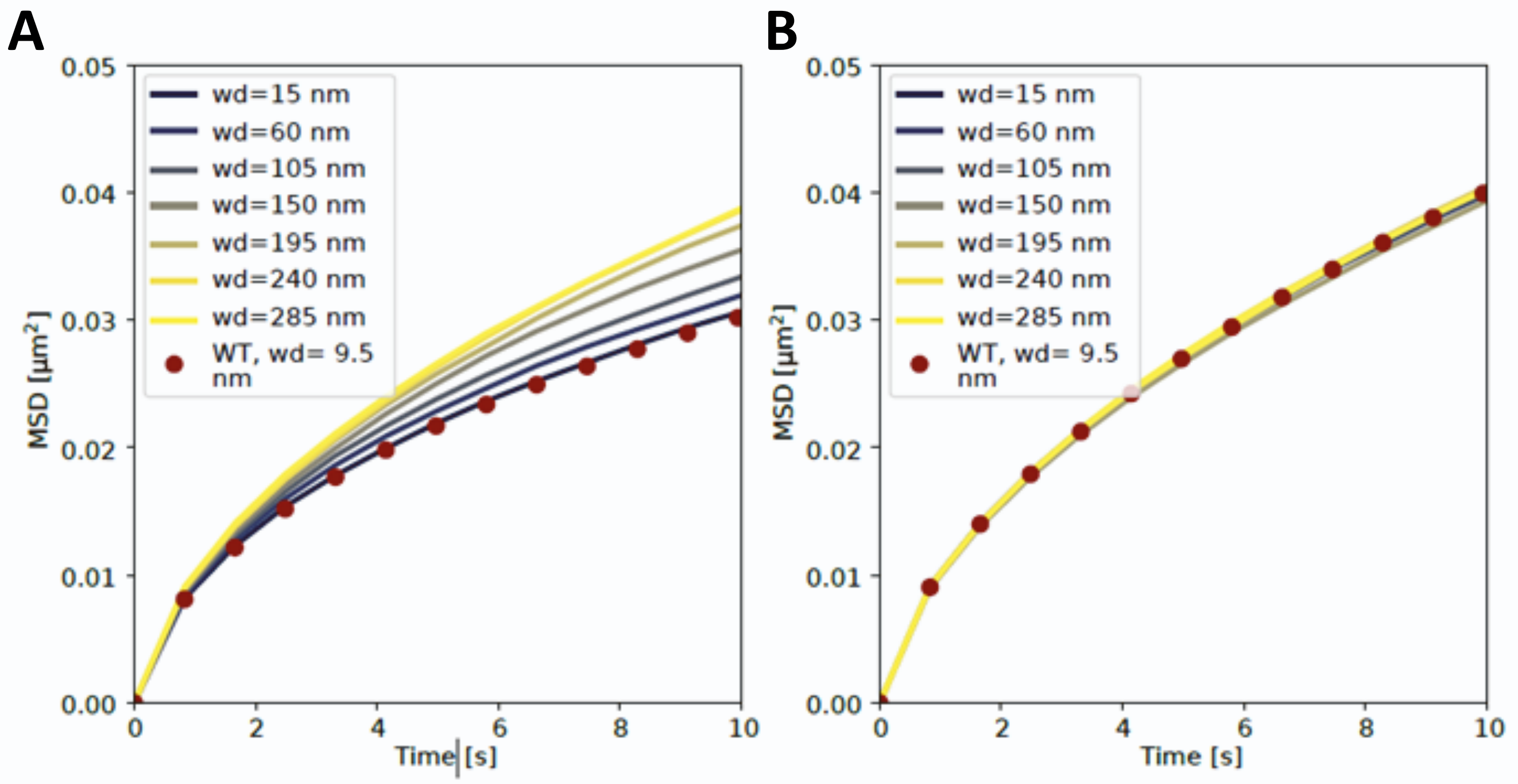

Figure S8

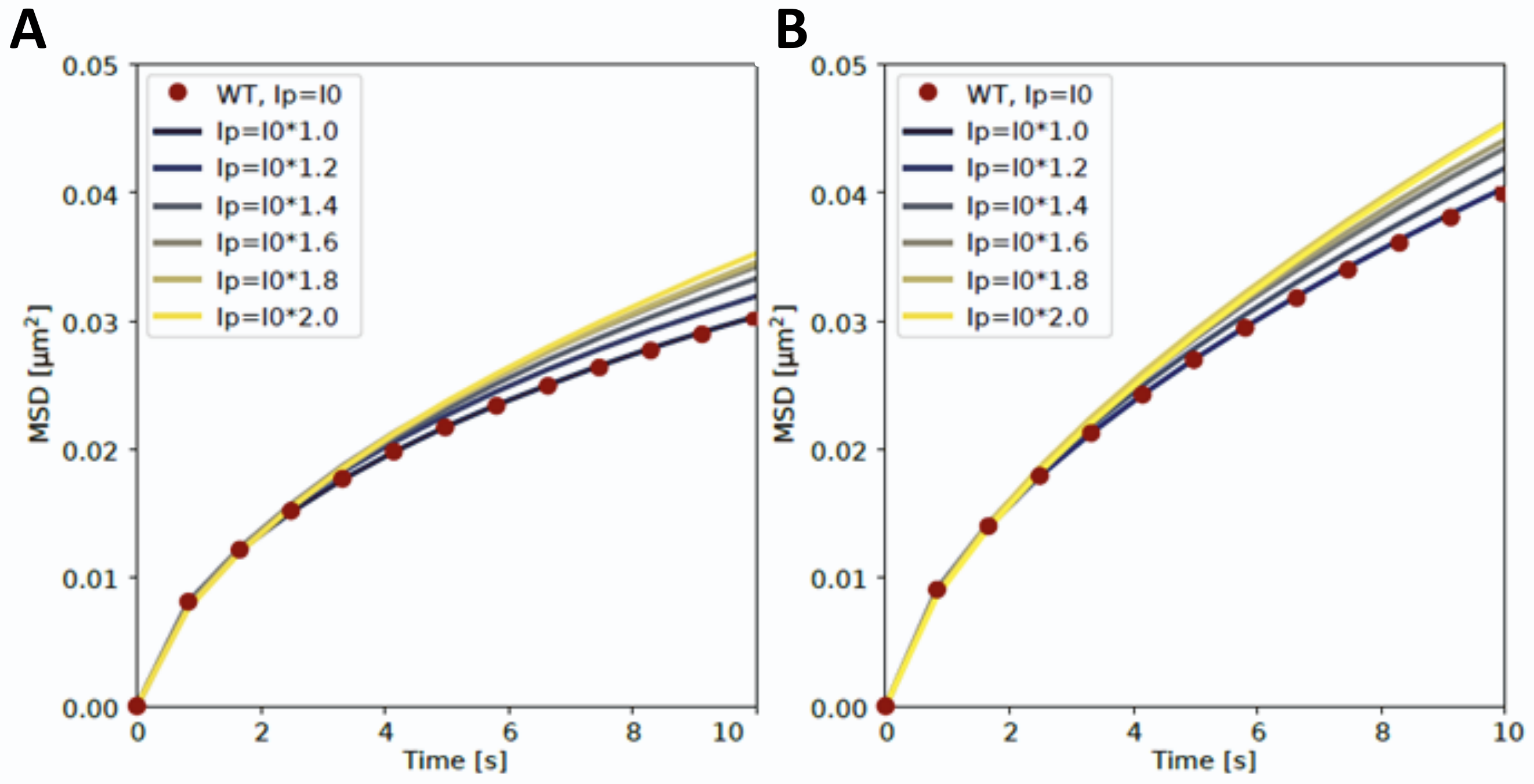

Figure S9

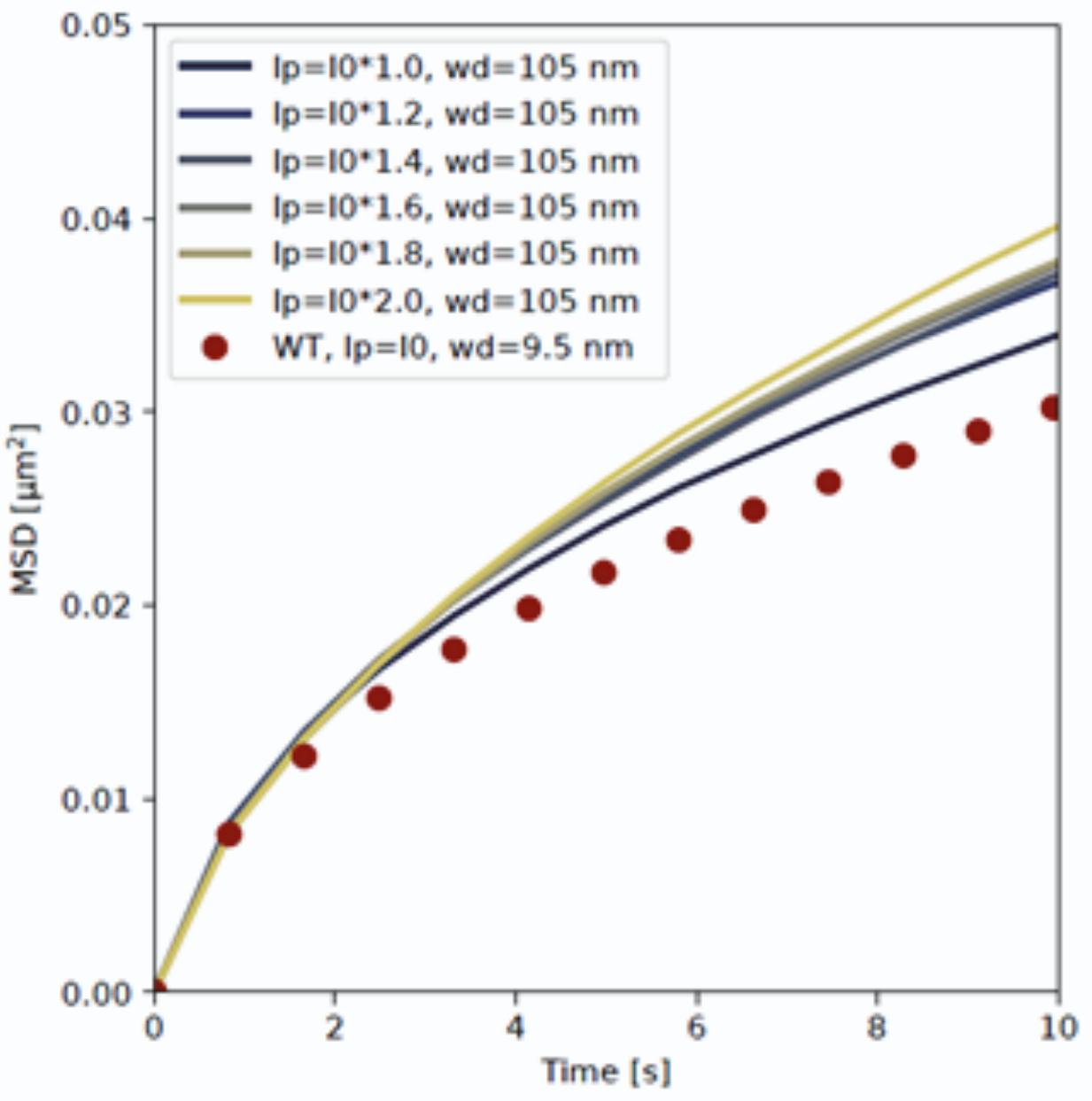

Ip=I0*1.0, wd=105 nm
Ip=I0*1.2, wd=105 nm
Ip=I0*1.4, wd=105 nm
Ip=I0*1.6, wd=105 nm
Ip=I0*1.8, wd=105 nm
Ip=I0*2.0, wd=105 nm
WT, Ip=I0, wd=9.5 nm
MSD [μm²]
Time [s]
0.00
0.01
0.02
0.03
0.04
0.05
0
2
4
6
8
10

Figure S10

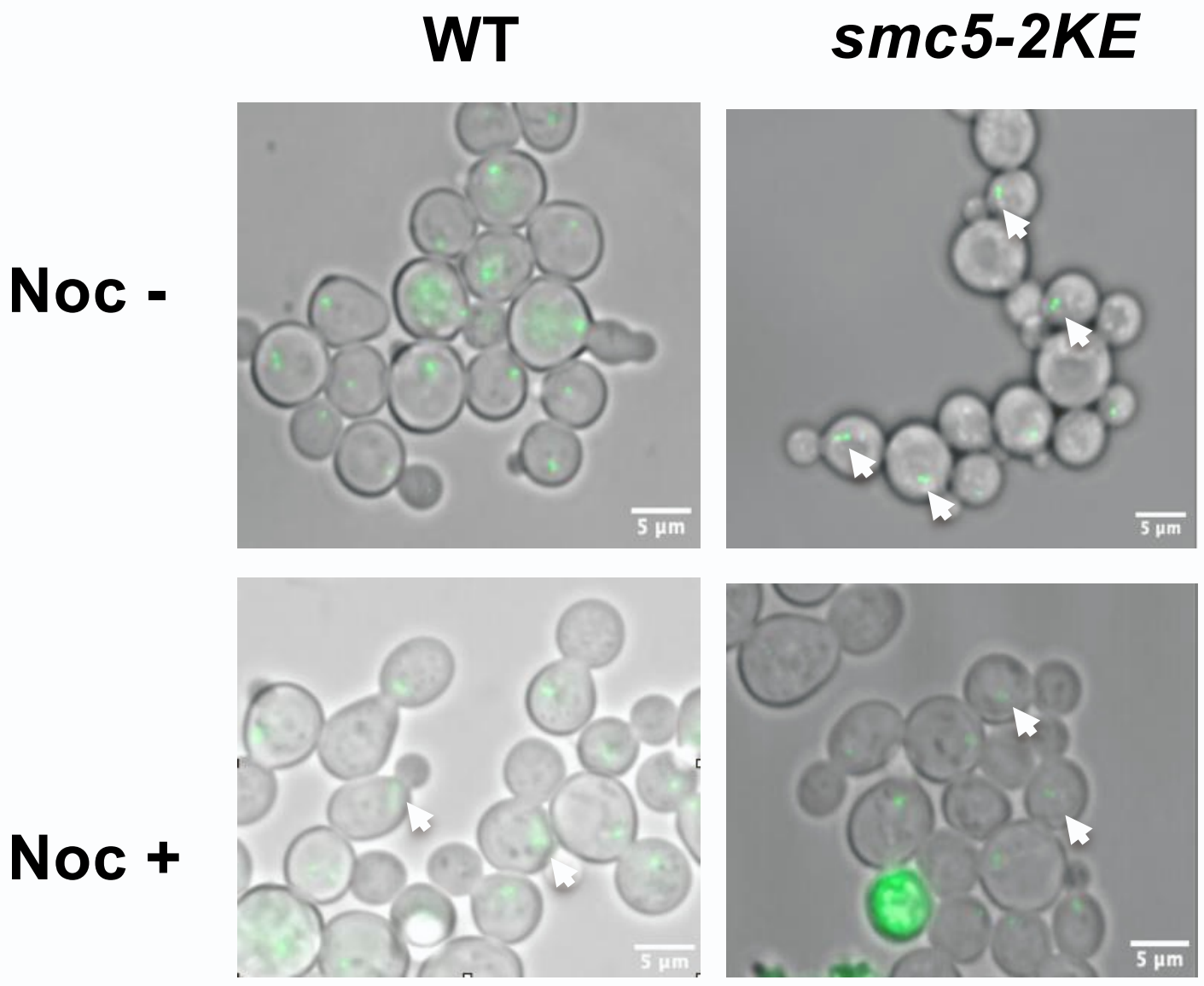

Figure S11

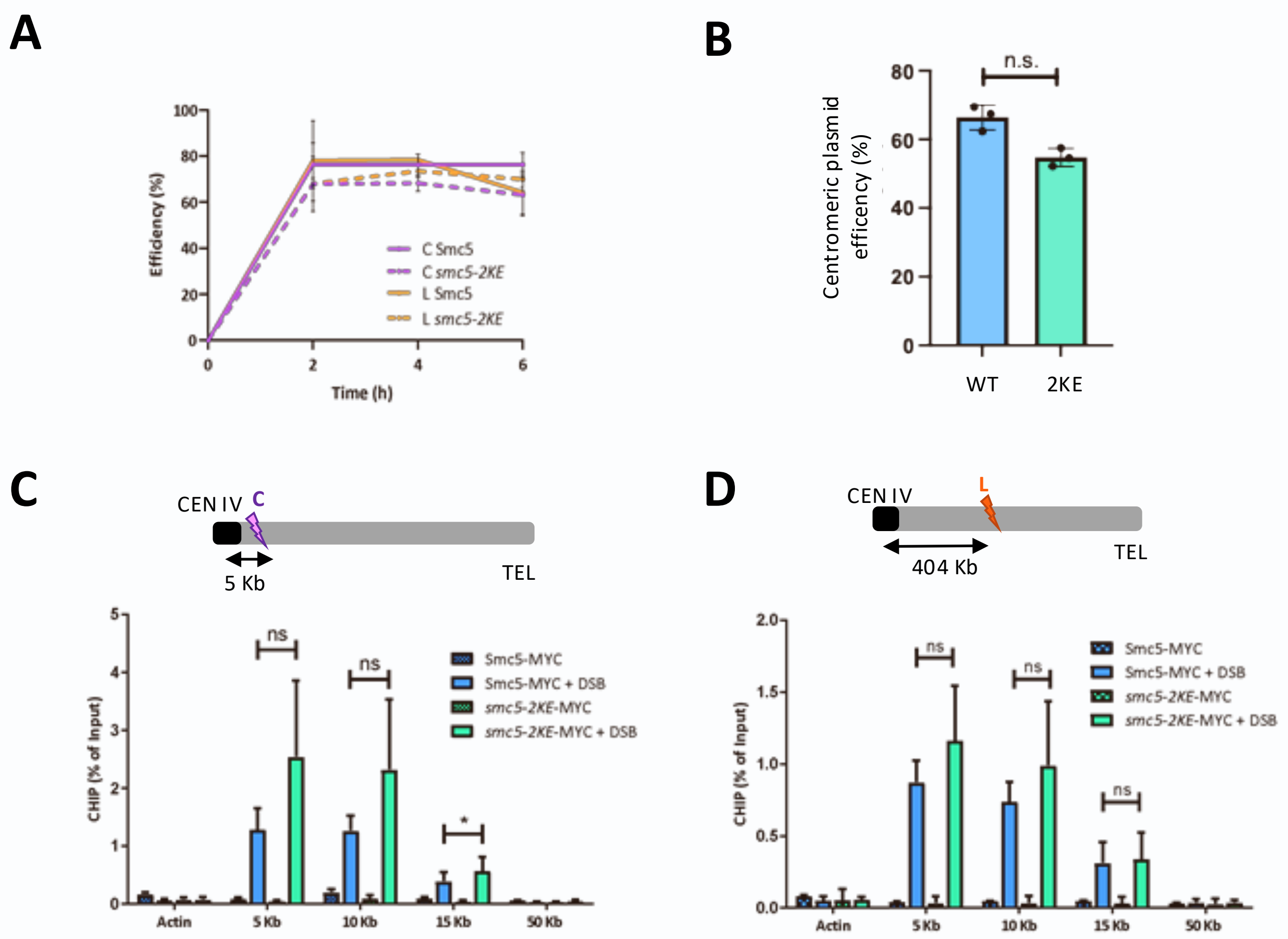
A
Efficiency (%)
Time (h)
C Smc5
C smc5-2KE
L Smc5
L smc5-2KE
B
Centromeric plasmid efficency (%)
n.s.
WT
2KE
C
CEN IV
C
5 Kb
TEL
CHIP (% of Input)
ns
ns
*
Smc5-MYC
Smc5-MYC + DSB
smc5-2KE-MYC
smc5-2KE-MYC + DSB
Actin
5 Kb
10 Kb
15 Kb
50 Kb
D
CEN IV
L
404 Kb
TEL
CHIP (% of Input)
ns
ns
ns
Smc5-MYC
Smc5-MYC + DSB
smc5-2KE-MYC
smc5-2KE-MYC + DSB
Actin
5 Kb
10 Kb
15 Kb
50 Kb

Table S1: primers used in this study

Gene / region	Primer	Sequence (5'□ 3')	Reference
**gRNA 2KE**	oACS13	CTGAAGGGCGAGATCAAGCgtttt	This study
	oACS14	CTCGTTGTGGGAGGTGATGTgatca	This study
**Donor 2KE**	oACS69	TCATGGGAATAGGTTAGTCGATATC**GAA**CAATCAGCATATGGTAGTgaaCAGGTCTTTCCTACTGACGTTA GTATTAAAC	This study
**Donor 2KA**	oACS97	TCATGGGAATAGGTTAGTCGATATC**GCG**CAATCAGCATATGGTAGT**GCG**CAAGTCTTTCCTACTGACGTT AGTATTAAAC	This study
**Donor 2KR**	oACS270	TCATGGGAATAGGTTAGTCGATATC**AGA**CAATCAGCATATGGTAGT**AGA**CAAGTCTTTCCTACTGACGTT AGTATTAAAC	This study
**Smc5-MYC**	oACS103	ATCCGAAGATGATACATTTCGGTGAAACTTCTAACTACTCATTCGAACGGATCCCCGGGttaattaa	This study
	oACS94	AAACATGTTTACATCTATATGTGTATAATTAATTATGCAATAGTGAAAGATGAATTCGAGCTCGTTTAAAC	This study
**AID**	oACS40	AGGATCCGAAGATGATACATTTCGGTGAAACTTCTAACTACTCATTCGATcgtacgctgcaggtcgac	This study
	oACS53	AAACATGTTTACATCTATATGTGTATAATTAATTATGCAATAGTGAAAGAatcgatgaattcgagctcg	This study
**AFB2**	oACS199	ACGCGCCGCTCACCCGCACGGCAGAGACCAATCAGTAAAAATCAACGGTTAATTCGGTCGAAAAAAGAAA	This study
	oACS200	AGCTTACATTTTATGTTAGCTGGTGGACTGACGCCAGAAAATGTTGGTGATTAGAGAATCCACACAAATG	This study
**KanMX R1**	OEA177	ACTTGGTTGGCGTGTTTCGT	This study
	OEA178	GCGGTTGTTTGCAAGACCGA	This study
**KanMX R2**	OEA181	GTCAAGGGCGTGTTTGACGC	This study
	OEA182	ACGAATTTTGCTCCGGCAATTCT	This study
**Cherry-*I-SceI*-cs**	OEA310	TAGGATACAGTTCTCACATCACATCCGAACATAAACAACCATGGTGAGCAAGGGCGA	This study
	OEA311	ATGACAAGTTCTTGAAAACAAGAATCTTTTTATTGTCAGTCTACTTGTACAGCTCGTCCATG	This study
**gRNA HIS3**	oACS13	GAGTGCGTTCAAGGCTCTTGgtttt	This study
	oACS14	CAAGAGCCTTGAACGCACTCgatca	This study
**Donor HIS3**	oACS69	GATTAACGTCCACACAGGTATAGGGTTTCTGGACCATATGATAT**AA**GCTCTGGCCAAGCATT CCGGCTGGTCGCTAATCG	This study
**R1 5 Kb**		GGCAATTGAGGCCTTCTGGC	García Fernandez et al., 2021
		TGGTGGCCAACGATCCTTCA	
**R1 10Kb**	oACS136	AGCGGAATCAGGAGTGCAAAA	This study
	oACS137	TGGAAAAATGCATCTACAACCGCT	
**R1 15 Kb**	oACS138	GCCAAAGGTGTTTTCATTTGGAAGG	This study
	oACS139	ACCAGCACCTGACTCTCCCA	
**R1 50 Kb**		TTCTCCCTTGTCACCGCCACC	García Fernandez et al., 2021
		GGCCGCTCAAGTTAACGCCCA	
**R1 150 Kb**		TGGGAATCGTTTTATCCTTGTTTCGC	García Fernandez et al., 2021
		GTGAAGCAACCAACATAATGCCAG	
	oACS194	ACGCGAGGATTTTTGTTCGTGG	

Table S2: plasmids used in this study

**Name**	**Marker**	**Features**	**Origin**
**pEF562**	KANMX	Plasmid carrying Cas9 and a gRNA scaffold. gRNAs were introduced by digesting this plasmid with BplI.	[87]
**pEF590**	KANMX	Plasmid coming from the pEF562 carrying the gRNA to cut close to the SMC5-K631.	This study
**pEF601**	URA3	Plasmid carrying pFA6a-13MYC-URA3 used to introduce 13x MYC at the C-terminus of *SMC5*	P. Lesage
**pEF222**	LEU2	Plasmid carrying pTEF-tTEF-LEU2-LacO, used to integrate the LacO in the place of a KanMX	Therizols et al., 2010
**pEF569**	HIS3	Plasmid carrying LacI-GFP-HIS3, which was linearized with *NheI* to introduce LacI-GFP into *HIS3*	García-Fernandez et al 2022
**pEF604**	HIS3	Plasmid carrying the AFB2, which was linearized with *NheI* to introduce AFB2-URA3 into *URA3* .	T. Teixeira
**pEF608**	HIS3	Plasmid coming from the pJH2970 (Addgene, 100954) carrying the gRNA to cut close to the Smc5-K631.	This study
**pB07**	HIS3	Plasmid carrying the I-*Sc*eI enzyme under GAL1-10 promoter	García-Fernandez et al 2022
**pEF573**	ADE2	Centromeric plasmid carrying the mCherry donor sequence, without the promoter and terminator.	García-Fernandez et al 2022
